# Plants Used by Bapedi Traditional Healers to Treat Asthma and Related Symptoms in Limpopo Province, South Africa

**DOI:** 10.1155/2018/2183705

**Published:** 2018-07-19

**Authors:** Sebua Silas Semenya, Alfred Maroyi

**Affiliations:** ^1^Technology Transfer Office, Research Administration and Development Department, University of Limpopo, Private Bag X1106, Sovenga 0727, South Africa; ^2^Medicinal Plants and Economic Development (MPED) Research Center, Department of Botany, University of Fort Hare, Private Bag X1314, Alice 5700, South Africa

## Abstract

To the best of our knowledge there are presently no ethnobotanical surveys focusing on the utilisation of herbal remedies for asthma in South Africa. The present study is therefore an attempt to fill this gap in knowledge. A total of 140 Bapedi traditional healers (THs) practicing in the Capricorn, Sekhukhune, and Waterberg districts of the Limpopo Province (South Africa) were queried using semistructured questionnaires, supplemented by field observations during face-to-face interview. A total of 104 medicinal plant species (92 indigenous and 12 exotics) belonging to 92 genera, distributed across 54 botanical families, mostly the Asteraceae and Fabaceae (18.5%, for each) as well as Malvaceae (12.9%), were used as antiasthmatics and related symptoms by these THs. Most of the plants were trees and herbs (37.5%, for each), with root (57%), leaf (15.8%), and bark (7.5%), respectively, being the saliently used parts for preparation of remedies.* Clerodendrum ternatum*,* Cryptocarya transvaalensis*,* Lasiosiphon caffer*,* Enicostema axillare*,* Mimusops obovata*,* Sclerocarya birrea*, and* Stylochaeton natalensis* were widely used and valued by all THs across the surveyed districts. Furthermore, these taxa also scored both the highest use value and fidelity level indexes as asthma therapies. Overall, the larger number of species documented in the present study is recorded for the first time in literature as asthma and/or related symptoms remedies. Our study finding generally contributes towards an establishment of South African database of herbal therapies used traditionally against these conditions.

## 1. Introduction

Asthma is a chronic lung disease that inflames and narrows the airways, affecting people of all ethnic groups worldwide [1]. Its symptoms include amongst others intermittent attacks of wheeze, cough, breathlessness with variable airway obstruction, chest tightness, and cough that occurs more at night and or early in the morning [2]. In 2008 at least three hundred million people worldwide were diagnosed with asthma and over 250,000 asthma-related annual deaths were reported [3].

Despite the above statistics, asthma continues to be a major world problem affecting people in various countries of the world including Australia [4], India [5], Jamaica [6], and Norway [7]. Health impact of this condition is also common in Africa, and studies conducted in Algeria [8], Nigeria [2], Uganda [9], and Zambia [10], amongst other countries, highlighted this. Asthma impact is also prevalent in South Africa. According to the recent report by the Global Initiative for Asthma, this country has the world's fourth highest asthma death rate amongst people aged five to 34 years [11]. Furthermore, of an estimated 3.9 million South Africans diagnosed with asthma, 1.5% die of this condition annually [12].

Treatment of asthma is focused on ad hoc treatment of acute exacerbations including lifestyle factors and prevention of exacerbations [13]. There are a number of top medications such as short or long acting beta_2_ agonist (preferably by inhalation) and inhaled steroid that an asthmatic patient can receive during exacerbations [3]. Supplementary medications for asthma sufferers include leukotriene receptor antagonists and theophylline or slow release beta_2_ agonist tablets [14]. However, these therapies are often limited and/or not affordable to a common man residing in most developing countries particularly in Africa [15, 16]. Consequently, asthma sufferers in these countries resort to locally available traditional healers (THs) who prescribe affordable herbal remedies.

There are extremely few ethnobotanical surveys reporting on the use of medicinal plants by indigenous people including THs as treatments of asthma and related symptoms in Africa as a continent. To the best of our knowledge the only studies that focused on this subject were conducted in Cameroon [17] and Nigeria [18]. However, general ethnobotanical studies carried out in other African countries, to name a few, Uganda [19], Kenya [20], Lesotho [21], and Botswana [22], highlighted that THs of other cultures do treat asthma and related conditions. South Africa is no exclusion and studies by Hutchings [23], Thring and Weitz [24], De Beer and Van Wyk [25], York et al. [26], and Bhat [27] also emphasised this. The present study therefore will be the first in South Africa to exclusively focus on ethnobotanical knowledge and practices of plants implicated in the treatment of these conditions.

## 2. Methodology

### 2.1. Study Area and Population

**Figure 1:**
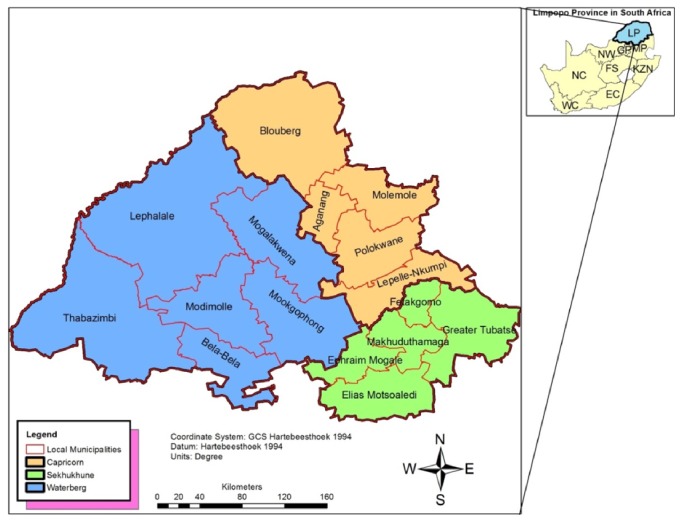
Map of Limpopo Province indicating the studied areas (districts and municipalities).

This study was carried out in the three districts (Capricorn, Sekhukhune, and Waterberg) of the Limpopo Province and associated municipalities (Figure 1).

A total of five rural villages from each municipality were chosen as study sites. In general, all these settlements are economically and socially marginalized [28]. Therefore, there is inadequate infrastructure, high unemployment, and dependency on natural resources amongst the people to support their livelihoods [28]. Furthermore, larger number of people still rely heavily on traditional methods of health care for treatment of various ailments [29]; thus THs and their services play an important role in the wellbeing of people. The Bapedi tribe who speak Sepedi language is the dominant ethnic group inhabiting the studied districts, representing more than 50% of the total population [30].

### 2.2. Ethnobotanical Survey and Data Collection

A reconnaissance study was firstly carried out in each selected village to (i) request permission from local tribal leaders to conduct this study within their areas of governance and (ii) ask THs who were conveniently (i.e., with the help of local leaders and healers) selected to participate in the survey. Both traditional leaders and healers were enlightened about the nature of the project including aim and objectives, using their mother tongue of Sepedi. Consequently, THs who agreed to take part in this study were requested to sign a consent form.

Data was collected from May 2017 to October 2017 using a semistructured interview with 140 THs during face-to-face interviews, supplemented by field trips for participant's observation and specimen collections. The questionnaire was designed to capture information on (i) local names of the plants used by Bapedi THs to manage asthma; (ii) plant parts used; (iii) state/s of used plant part; and (iv) mode/s of preparation and administration of remedies. Overall, THs were questioned independently in their consultation rooms using Sepedi dialect.

Field excursions for medicinal plant species identification and collection were conducted with the assistance of each questioned traditional healer. During these trips THs initially identified the species via vernacular names. Subsequently, voucher specimens were collected, prepared, and deposited at the Larry Leach Herbarium (University of Limpopo), wherein a scientific name of plant species was established by a trained taxonomist.

### 2.3. Data Analysis

#### 2.3.1. Microsoft Excel and Statistical Package for the Social Sciences (SPSS)

The data collected in this study were analysed using Microsoft Excel 2000 and SPSS version 14.0. Descriptive statistics using frequencies and cross-tabulations were utilised in constructing tables showing the commonly used plant species by THs, local names of the plants used for asthma and related conditions, plant parts used and state/s of their usage, modes of preparation, and remedy administration.

#### 2.3.2. Fidelity Level (FL)

The FL as described by Al-Quran [31] were used to determine the uniformity of plant utilisation amongst the questioned THs. Analysis of FL of each plant species mentioned by Bapedi THs as a treatment of asthma and related symptom followed the formula displayed below:

(1) $$\begin{array}{c} FL\left(\;\%\;\right)=\frac{NP}{N}\times 100, \end{array}$$

where *Np* was the number of THs who claim the use of a particular plant species to treat asthma or related symptom and *N* was the total number of THs who mentioned the use of species as a medicine to treat any given ailment/s (asthma or related symptom). Fidelity level expresses the preference a species is given over others in the management of a particular ailment [32].

#### 2.3.3. Use Value (UV)

Use values are calculated for an individual plant, in order to objectively give a quantitative measure of its relative importance to the informants [33]. Therefore, the extent of utilisation of each species used therapeutically by Bapedi THs for asthma and related symptoms was determined via UV, following Phillips and Gentry [33] index:

(2) $$\begin{array}{c} UV=\sum \frac{U}{N} \end{array}$$

From the above formulation, U was the number of curative applications of each species, where *N* represented the total number of THs. Generally plant with broad therapeutic uses or those that are highly accepted as cure of a particular ailment will score a high UV.

## 3. Results and Discussions

### 3.1. Diversity of Used Plant Species

This is the first study of its kind in South Africa and few of those conducted in other African countries [17, 18] and elsewhere [34] that focused on the utilisation of plants to treat asthma and related symptoms. A total of 104 medicinal plant species (92 indigenous and 12 exotics) belonging to 92 genera, distributed across 54 botanical families, mostly the Asteraceae and Fabaceae (n=10 spp., for each, 18.5%), Malvaceae (n=7 spp., 12.9%), Anacardiaceae, and Euphorbiaceae (n=4 spp., for each, 7.4%), respectively, were recorded as being used by 140 Bapedi THs to treat these conditions. This diversity of plants is higher compared to 46 noted by Sonibare and Gbile [18] in Nigeria, and 29 by Noumi [17] in Cameron. The observed variation might be attributed to extremely larger sample size of THs and spatial coverage included in our study compared to these two studies. Some of the above-mentioned botanical families documented in our study are repeatedly noted as predominant in various ethnobotanical surveys focusing on asthma. For instance, the Asteraceae, Euphorbiaceae, and Fabaceae were also represented with higher number of species in a study conducted in India [34]. In a similar survey carried out amongst THs in South Western Nigeria, Euphorbiaceae was also dominant [18]. The widespread and higher utilisation of species from all the aforesaid botanical families is an indication that they are widely distributed in various countries of the world. Overall, their high preponderance in the present study might be due to the fact that they contain a relatively higher integer of antiasthmatic taxa locally known by Bapedi THs compared to the rest of plant families which had less than four taxa (Table 1).

### 3.2. Plant Habit

Plants documented in this study were mainly trees and herbs (n=39, for each) as well as shrubs (n=26). This finding, however, is not surprising because these growth forms are prevailing components of local flora distributed across the studied districts and municipalities. According to Shankar et al. [35] the more common the growth form is in an area, the greater the probability of its popular use is. Therefore, Bapedi THs might prefer the aforesaid habits due to their local availability and familiarity.

### 3.3. Distribution of Used Plants within the Municipalities and Districts

The recorded 104 plant species were not used by all THs who diagnosed asthma and its symptoms across the studied municipalities and districts. Overall the most widely distributed medicinal plant species (6.7%, n=7) used in all these geographical areas were* Clerodendrum ternatum*,* Cryptocarya transvaalensis*,* Lasiosiphon caffer*,* Enicostema axillare*,* Mimusops obovata*,* Sclerocarya birrea*, and* Stylochaeton natalensis*. The widespread utilisation of these species shows that they are popular, safe, and well-known as asthma therapies in Bapedi traditional healing sectors.

An overwhelming majority (40%, n=42) of species comprising* Abutilon galpinii, Acacia erioloba, Acacia senegal, Acacia sieberiana, Albizia adianthifolia, Allium sativum, Aloe falcata, Berchemia discolor, Blepharis diversispina, Blepharis subvolubilis, Buddleja salviifolia, Cassinopsis ilicifolia, Catha edulis, Clivia caulescens, Dicerocaryum senecioides, Dicoma anomala, Dodonaea viscosa, Dombeya rotundifolia, Elephantorrhiza goetzei, Eucalyptus camaldulensis, Grewia hispida, Grewia sulcata, Harpephyllum caffrum, Helichrysum caespititium, Helichrysum gymnocomum, Hypoxis obtusa, Ipomoea albivenia, Jatropha zeyheri, Mangifera indica, Opuntia ficus-indica, Panica granatum, Pyrenacantha grandiflora, Rhoicissus tomentosa, Schkuhria pinnata, Sida cordifolia, Siphonochilus aethiopicus, Sorghum bicolor, Tragia dioica, Tulbaghia violacea, Zantedeschia aethiopica, Zea mays,* and* Ziziphus mucronata* were used in a single municipality located within one of the three studied districts (Table 2). This finding might be attributed to the natural distribution of these taxa in the studied districts or variation with respect to THs' indigenous knowledge related to their applications as antiasthmatic medicines.

The above can also be said for 19.3% (n=20) of plants, namely,* Adansonia digitata, Adenia fruticosa, Aloe *spp*., Citrus limon, Clerodendrum glabrum, Cucumis metuliferus, Englerophytum magalismontanum, Erythrina lysistemon, Eucomis autumnalis, Euphorbia schinzii, Lantana rugosa, Leonotis leonurus, Maerua juncea, Olea europaea, Pseudognaphalium luteo-album, Solanum catombelense, Solanum panduriforme, Vepris reflexa, Warburgia salutaris,* and* Xerophyta retinervis,* which were utilised by THs in particular municipalities located in one district. The remainder (33.7%, n=35) of the plant species recorded in the present study were also distributed in certain municipalities but in more than one district (Table 2).

### 3.4. Species Utilisation and Literature Comparison

Amongst the 104 plants recorded in the current study, vast majority (50.9%, n=53) were used by THs to exclusively manage asthma, and 25.9% (n=27) for both asthma and the following symptoms: fatigue, nasal congestion, tight chest, wheeze, laboured breathing, nasal congestion and wheezing, fatigue and wheezing, and fatigue and laboured breathing, as well as fatigue, laboured breathing, nasal congestion, and wheeze. The remaining 24.0% (n=25) of the taxa were used exclusively to heal some of these symptoms (Table 1). Overall all taxa recorded in this study are known medicinal plants in South Africa and thus form part of local traditional ethnopharmacopoeia of various cultures in this country. However, the practice of Bapedi traditional healers to select various plant species to exclusively treat asthma might be attributed to a number of factors including the availability of some plants during certain season of the year and in selected geographical areas. Furthermore, it is perhaps a means to allow them to select more effective asthma remedy or it is traditional practice they learned from their mentors.

#### 3.4.1. Asthma Therapies

Of the 53 (50.9%) asthmatic species, six (11.3%) comprising* C. ternatum*,* C. transvaalensis*,* E. axillare, M. obovata*,* S. birrea*, and* S. natalensis* were appreciated by all THs (n=140) who treated asthma across the study sites. To the best of our knowledge, with the exclusion of* S. birrea *which were previously highlighted by Ojewole [36] as being used for asthma in unspecified Southern Africa countries, all the aforesaid taxa are recorded for the first time in our study as remedy for this ailment. However, such species are well-known and widely used as traditional cure for other human diseases across Africa. Hossan et al. [37] observed that medicinal plants that are both highly and widely used for a particular ailment are in most case new sources of medication of such affliction. Taking into account this, we hypothesize that the wide use of the above-listed six taxa in the treatment of asthma by Bapedi THs is due to their effectiveness and thus must be investigated for their potential as new source of asthma medication. In fact utilisation of* E. axillare *by these THs is already supported by scientific studies. For instance, its extract exhibited both anti-inflammatory and antiasthmatic activities [38]. Ethanol extracts of* Clerodendrum serratum* Linn roots showed good antiasthmatic activity in experimental animal [39], thus suggesting that a closely related species* C. ternatum *(used by Bapedi) might also possess same activity.

Most (50%, n=26) of the remaining plants,* A. galpinii, A. erioloba, A. senegal, A. sieberiana, B. discolor, B. salviifolia, C. edulis, C. glabrum, C. caulescens, C. metuliferus, D. senecioides, Dioscorea sylvatica, E. magalismontanum, E. schinzii, G. sulcata, H. caffrum, I. albivenia, J. zeyheri, L. rugosa, P. grandiflora, R. tomentosa, S. pinnata, Senna italica, S. catombelense, S. panduriforme,* and* Strophanthus speciosus,* used in this study to exclusively treat asthma were also documented for the first time in this study as remedies for this condition. These species are also traditionally utilised as medicine to treat different human diseases in South Africa and other African countries. Therefore, their use in this study for asthma is an indication that they might be safe for consumption as remedies. From conservation point of view restricted knowledge of the above-mentioned species to Bapedi THs as therapies for asthma to some extent has advantage, as it decreases the impact of being extensively and recurrently harvested across the countries to manage this chronic disorder.

The utilisation of the rest (38.5%, n=20) of the species, namely,* Alepidea amatymbica, Cassia abbreviata, Carpobrotus edulis, D. anomala, E. camaldulensis, Gossypium herbaceum, H. gymnocomum, L. leonurus, M. indica, O. europaea, O. ficus-indica, P. granatum, S. cordifolia, S. aethiopicus, Securidaca longepedunculata, Ximenia caffra, X. retinervis, Z. aethiopica, Z. mucronata,* and* Z. mays,* used for asthma by Bapedi THs was previously culturally validated either in South Africa, in other African countries, or elsewhere. Nonjinge and Tarr [40] who worked with Zulu THs of KwaZulu-Natal Province noted* A. amatymbica* as a valued medicine for asthma. In other studies conducted in Zimbabwe [41, 42], this species was amongst the ten most used asthma remedies. However, in the present study,* A. amatymbica* was only used by 7.8% (n=11) of all 58.3% (n=140) THs who treated asthma, which might be attributed to its rare status across the country [43]. In view of this and the fact that asthma is a chronic disorder requiring a readily available medicine for its management, most THs in this study might have less preferred and considered* A. amatymbica* therapies as unsustainable.

The knowledge of* D*.* anomala* use by Bapedi THs to treat asthma is supported by finding of Van der Merwe [44] who worked with Zulu THs. In fact most of the previously ethnobotanically validated asthmatic species used in the current study corroborate with those used by Zulu, compared to with other cultures.

Use of* L. leonurus* as recorded in this study was previously noted by Hutchings et al. [23] who questioned Zulu THs and Nzue [45] who worked with Rastafarians of Western Cape Province of South Africa. Similar finding was reported amongst Swati THs residing in Swaziland [46]. The observed similarities regarding the application of* L*.* leonurus* amongst South African and Swazi healers might be due to a cross-border transfer/exchange of knowledge. This posit is ascribed to the fact that most of Swaziland is locked within South Africa, which might had allowed easy transfers of knowledge amongst THs across geographical borders.

Similarly to Bapedi THs, Zulu also use* S. aethiopicus *as asthma medicine [23]. Widespread use of this species in the treatment of respiratory infections including asthma by Zulu THs has wiped out its entire local population within the communal lands in KwaZulu-Natal Province [47]. However, in the present study extent of use of* S. aethiopicus *specifically for asthma might currently not have profound impact on reduction of its natural population based on the fact that it is only used by 0.7% (n=1) and also in combination with other species, which both put less harvesting pressure on the population.

The utilisation of* X*.* retinervis *[48] and* Z*.* mucronata *[49] by Bapedi THs in the treatment of asthma was previously highlighted by the mentioned authors amongst the unspecified South African ethnic groups. Extracts of* X*.* retinervis *[50] and* Z*.* mucronata *[51] were active against pathogens causing respiratory infections, which may possibly indicate that they might be helpful in the management of asthma or related symptoms.

With the exclusion of* Z. aethiopica *which is also used as medicine for asthma by the Xhosa people of South Africa [52], the remaining species, namely,* E. camaldulensis*,* M. indica*,* O. ficus-indica*,* P. granatum*,* S. longepedunculata*,* X. caffra*, and* Z. mays*, used exclusively by Bapedi for this condition, are recorded for the first time in South Africa as asthma therapies. However, their use in the treatment and management of this condition is common in other African countries or elsewhere. For instance, Nigerian THs also use* E. camaldulensis *[53],* M. indica *[54], and* S. longepedunculata* [55] to treat asthma. Comparably to our findings, Naoumi [17] reported the use of* M*.* indica *and* Z*.* mays* as medicines for this ailment by THs in Cameroon. Utilisation of* X. caffra *as asthma therapy is also common in Swaziland [56]. These findings support the general notion that Africans share the same indigenous knowledge [57].

To the best of our knowledge ethnobotanical records regarding uses of* G. herbaceum*,* O. europaea*,* O. ficus-indica*, and* P. granatum* in the management of asthma are nonexistent in Africa, thus noted in this study for the first time. However, the taxa* G. herbaceum *[58],* O. europaea* [59],* O. ficus-indica* [60], and* P. granatum* [61] are all used in other continents of the world comparably to Bapedi THs, subsequently, indicating that these species might be helpful as asthma remedies. Some of the aforementioned taxa, notably* E*.* camaldulensis*,* M*.* indica*,* O*.* ficus*-*indica*,* P*.* granatum*, and* Z*.* mays*, are exotic in South Africa, thus suggesting two things: (i) that the original knowledge of their application for asthma by Bapedi was obtained via interactions with outside THs and/or (ii) was given by ancestors via dreams. The last posit is based on the fact that most of interviewed THs claimed that their ancestors show them new uses of medicinal plants via dreams while asleep. In general, fruits of* M*.* indica*,* O. europaea*,* O*.* ficus*-*indica*,* P*.* granatum*,* X. caffra*, and* Z*.* mays* were stated by THs as also being harvested for household consumption. Therefore an investigation into the potential of fruits from these species as asthma therapies will be interesting, and if effective it should be manufactured as beverages that assist in the asthma management. Ethanol extracts (100 mg/kg, p.o.) of* P. granatum* [61] and aqueous extract of* O. europaea* [62] fruits have already demonstrated a significant antiasthmatic activity at experimental model [61].

#### 3.4.2. Asthma and Related Symptoms Therapies

As noted earlier, 25.9% (n=27) of species were multiused by THs to treat asthma and the following symptoms: fatigue, nasal congestion, tight chest, wheeze, laboured breathing, nasal congestion and wheezing, fatigue and wheezing, and fatigue and laboured breathing, as well as fatigue, laboured breathing, nasal congestion, and wheeze (Table 1). Amongst these plants, 37% (n=10) comprising* Aloe* spp.,* A. fruticosa*,* Adenia spinosa*,* Callilepis laureola*,* Cyperus sexangularis*,* Elephantorrhiza burkei*,* Hypoxis hemerocallidea*,* M. juncea*,* Peltophorum africanum,* and* Protea caffra* were stated by THs as cure for asthma and fatigue. Of these taxa only use of* H. hemerocallidea* for asthma [63] and fatigue [64], as well as* P. africanum *for the latter condition [65], was previously reported in ethnobotanical literature. Use of* H. hemerocallidea* to cure fatigue by Bapedi THs was expected mainly due to its popularity as effective energy-booster. For instance, in almost every pharmaceutical chemist in Limpopo Province, there are various scientifically authenticated herbal formulations (e.g., Hypo-Plus®) made from* H. hemerocallidea* [66], which are being advertised on local radios and newspapers as effective energy and immune boosters. Thus, Bapedi THs might have had a talk about this and decided to include* H. hemerocallidea* as part of their fatigue treatment in asthma sufferers. On the other hand, use of this species as antiasthma by Bapedi THs might be due to its efficacy in the management of asthma and related conditions, attributed to its antiinflammatory activity [67]. The use* A. fruticosa*,* A. spinosa*,* C. laureola*,* C. abbreviata*,* C. sexangularis*,* E. burkei*,* M. juncea*,* P. caffra *(asthma and fatigue), and* P. africanum *(asthma) as therapies for the mentioned aliments as disclosed by Bapedi THs was not found in literature, thus reported in the present study for the first time.

Species used as medicine for asthma and nasal congestion made up 18.5% (n=5) and included* K. wilmsii*,* P. punctulata*,* P. zeylanica*, S*. nitidum*, and* V. natalensis*. Amongst these species only* P. zeylanica *[68] and* P. punctulata *[69] were previously recorded in literature as asthma treatment but no records of its applications for nasal congestion exist. Restricted uses of* K*.* wilmsii* to Bapedi THs as medicine for these illnesses might be due to the fact that it is localised in the Capricorn and Sekhukhune districts (Limpopo Province) both mainly inhabited by the Bapedi culture. This might be true since the known general medicinal usage of* K*.* wilmsii* is presently restricted to this culture.

Only 7.4% (n=2) of species* C. sativa* and* L. caffer* were used to heal asthma and tight chest in this study. Utilisation of* C. sativa *as asthma medication was previously noted by Van Wyk and Gericke [70] amongst the unspecified South African ethnic groups. Its use for tight chest is recorded in our study for the first time in African ethnobotanical literature. However,* C. sativa *is commonly used for this condition by THs in Pakistan [71]. Its restricted uses for tight chest to Bapedi THs across Africa might somewhat be attributed to the fact that it is a legally declared drug; thus any person who is found in its possession without a permit is prosecuted. In fear of this most THs might retaliate to divulge its uses to researchers. No ethnobotanical record of* L. caffer* as treatment of asthma and tight chest was found in literature. However, this species was used by all interviewed Bapedi THs (n=140) as cure for asthma, which might be a reflection of its bioactivity against this condition.

Asthma and wheeze were also treated with two (7.4%) species, namely,* W. salutaris* and* Z. capense*. Amongst these trees only use of* W. salutaris* as antiasthma was previously reported in literature [23, 71]. The remainder of documented uses of both the aforesaid trees is reported for the first time in this study. However, lack of literature based information regarding their use for wheeze is understandable, based on the fact that this condition is one of the key symptoms of asthma. Consequently, THs of other cultures might have realised that a successful asthma treatment or management with* W. salutaris* and* Z. capense* automatically addresses all symptoms. It is also possible that use of these species by Bapedi for wheeze has specific impact on reducing constriction in the airways, and thus contributing towards reduction of wheeze sound.

Species used in the present study for asthma and laboured breathing were only (3.7%, n=1)* A. sativum*. The stated uses of this species are recorded in our study for the first time in South Africa but are common in other countries. For instance, its use as asthma medicine was previously reported in Egypt [72] and Nigeria [73]. However, as far as our literature search went, application of* A. sativum *for laboured breathing is presently restricted to Bapedi THs in Africa but used as such by THs in India [74]. Limited use of this species amongst indigenous people of South Africa might be due to the fact that it is mostly found in the markets. Thus in view of chronic nature of asthma and lack of income to frequently purchase its material, native people might have opted for an alternative species available in free access communal lands. Few (n=2) of Bapedi THs who use* A. sativum *in the present study harvest it from home gardens.

Asthma, nasal congestion, and wheezing were treated with two (7.4%, n=2) aromatic species* A*.* afra* and* C. gratissimus*. Utilisation of both species for wheezing is currently restricted to the Bapedi THs. However, our finding regarding use of* A*.* afra* in the treatment of asthma and nasal congestion coincides with that reported by Mukinda [75] amongst Xhosa THs of the Western Cape Province (South Africa). Similarly, application of* C. gratissimus* for asthma as noted in the present study was previously highlighted by Morobe et al. [76] in South Africa. No previous record of* C. gratissimus* as nasal congestion remedy was found in literature; thus it is reported for the first time in the present survey.

A total of two (7.4%) species* D. elata* and S.* serratuloides* were multiused by Bapedi THs to cure asthma, fatigue, and wheezing. Only use of* D. elata *as medicine for the first condition was previously highlighted in ethnobotanical literature [77]. The remainder of the applications of aforesaid species is currently restricted to Bapedi THs. Anti-inflammatory properties of S.* serratuloides* were reported by Fawole et al. [78], therefore suggesting that its use for asthma and related condition in the present study might be effective.

Another 7.4% (n=2) of species (*Schinus molle* and* O. lanceolata*) were multiused by Bapedi THs for asthma, fatigue, and laboured breathing. With the exclusion of using an exotic* S. molle* as asthma medication which was culturally validated in Peru [79], application of the rest of species is reported for the first time in this study across South Africa and Africa as a continent.

Overall an aromatic herb* L. javanica *was the only (3.7%, n=1) species widely used by Bapedi THs. For instance, it was multiused as medicine to heal asthma, fatigue, laboured breathing, nasal congestion, and wheeze. Use of* L. javanica *to treat asthma [80], fatigue, and nasal congestion [26] as well as laboured breathing [81] is common amongst other South African cultures. However, its use for wheeze is presently restricted to the Bapedi THs. In general, wide usage of* L. javanica *for asthma and perceived related symptoms by these THs might be ascribed to its wide local abundance across the Limpopo Province, and its popularity as treatment of respiratory infections [51].

#### 3.4.3. Therapies for Asthma Symptoms

The rest (24.0%, n=25) of the species recorded in this study as part of asthma management were exclusively used by THs to treat various conditions they perceived as being associated with this inflammatory condition (Table 1). Amongst these plants 88% (n=22) were used to treat a single ailment, namely, fatigue (*A. digitata*,* A. adianthifolia*,* A. falcata*,* B. diversispina*,* B. subvolubilis*,* C. edulis*,* C. ilicifolia*,* D. rotundifolia*,* E. goetzei*,* H. caespititium*,* H. obtusa*,* M. oleifera*,* P*.* luteo*-*album*,* S. bicolor*,* T. dioica, *and* V. reflexa*), laboured breathing (*D. viscosa*), nasal congestion (*G. hispida*,* K*.* brachyloba, *and* T. violacea*), tight chest (*C. limon*), and wheezing (*E. lysistemon*). The remainder (12%, n=3) of the species, namely,* E. autumnalis *(fatigue, nasal congestion),* E. pallidiflora* (fatigue, nasal congestion, and wheeze), and* W*.* somnifera* (fatigue and laboured breathing) were multiused. Overall, applications of an overwhelming majority of the above-listed species by THs are recorded for the first time in this study. For instance, with the exclusion of* A. digitata* [82],* C. edulis* [83],* M. oleifera* [84], and* W*.* somnifera* [85, 86], which their utilisation as mentioned by Bapedi was previously highlighted in African literature, use/s of the remaining species are currently restricted to Bapedi THs. Overall, a larger number of the above-mentioned new medicinal use of commonly known species by Bapedi THs would let one believe that they are still experimenting or further exploring other potential uses of local flora with the hope of discovering new effective plants that could contribute towards the wellbeing of asthmatic patients.

### 3.5. Fidelity Level (FL) and Use Value (UV)

Fidelity levels of the recorded plant species differed widely for specific disease/s. The maximum fidelity level of 100% was reported for 71.1% (n=74) of species, with the majority having extremely lower use-mention (UM) against a particular ailment (Table 1). Indeed Ong and Kim [87], stated that high FL can only imply that a particular plant is most preferred if there is considerable number of use-mentions from participants. Therefore, we have correlated FL and UM in order to establish the accurate FL of each species. In this regard, species with 100% FL coupled with use mentioned of less than 15 times were not considered. Accordingly,* S. birrea* (UM = 140 and FL = 100; asthma),* S. natalensis *(UM = 140 and FL = 100; asthma),* E. axillare *(UM = 140 and FL = 100; asthma),* C. ternatum *(UM = 140 and FL = 100; asthma),* C. transvaalensis *(UM = 140 and FL = 100; asthma),* M. obovata *(UM = 140 and FL = 100; asthma),* L. caffer *(UM = 140 and FL = 100; asthma, and UM = 17 and FL = 10.8; tight chest)*, C. sexangularis *(UM = 58 and FL = 89.2; fatigue),* A. digitata *(UM = 68 and FL= 100; fatigue),* G. herbaceum *(UM = 70 and FL= 100; asthma),* A. afra *(UM = 25 and FL = 75.7%; asthma),* A. spinosa *(UM = 25 and FL = 30.4; asthma and UM = 57 and FLl = 69.5; fatigue),* P. obliquum *(UM = 25 and FL = 100),* E. schinzii* (UM = 19 and FL = 100; asthma),* M. oleifera *(UM = 17 and FL = 100; fatigue), and* S. speciosus *(UM = 15 and FL = 100; asthma), respectively, scored the highest FL amongst the plants used by Bapedi THs for asthma and related symptoms, thus suggesting their potential as therapies against the noted corresponding specific conditions.* Adenia spinosa *and* L. caffer* which were used to treat two conditions could be of great importance in the management of various ailments.

Relatively high UVs was observed for* L. caffer *(UV = 1.2; asthma and tight chest),* M. obovata *(UV = 1; asthma),* C. ternatum *(UV = 1; asthma),* C. transvaalensis *(UV = 1; asthma),* E. axillare *(UV = 1; asthma),* S. natalensis *(UV = 1; asthma), and* S. birrea *(UV = 1; asthma). As noted earlier all these species exhibited maximum (100%) FL as antiasthmatics therapies; thus their highest UV for similar treatment further accentuates their prospective in the management of asthma.

### 3.6. Plant Parts Used, Mode of Preparations, Dosages, and Administrations

The majority of the herbal medicines used by Bapedi THs as asthma and related symptoms therapies were mainly prepared from root (57%, n=61), leaf (15.8%, n=17), bark (7.5%, n=8), bulb and whole plant (5.6%, n=6, for each), fruit and tuber (2.8%, n=3 for each), seed, stem, and rhizome (0.9%, n=1, for each), respectively. Three species,* L. leonurus* (root and leaf),* S. birrea* (bark and fruit), and* P. africanum* (bark and root), were harvested for their two different parts. Contrary to the results of the present study, Sonibare and Gbile [18] found that THs in Nigeria prefer stem bark to make asthma remedies. Extensive use of root in this study was based on the perception that it carries more healing power as opposed to other plant parts, a finding which was previously reported by Semenya [88], who worked with Bapedi THs. Indeed it has been scientifically demonstrated that plant root contains many bioactive principles [88]. However, extensive exploitation of roots by these THs should proceed with caution as it might endanger the species. Higher usage of leaves by Bapedi THs might be linked to their ease of collection and transportation, both compared with other parts.

The above-mentioned plant parts (n=107) used for herbal preparation were mostly processed by Bapedi THs in their dried states (78.5%, n=84) than when they are fresh (21.4%, n=23). This finding might be attributed to the fact that these THs preserve most of their medicine in dried form for future uses.* Sclerocarya birrea* was processed in both dried and fresh states. Overall, a total of 153 recipes were used by Bapedi THs to treat asthma. Of these formulae, monotherapies (75.1%, no = 115) based on a single plant preparation were dominant. A similar finding was noted by Noumi [17] in Cameroon. On the contrary Sonibare and Gbile [18] found that more of herbal medicine prescribed by THs in Nigeria are made from more than one species (multitherapies) in Nigeria. High use of monotherapies by Bapedi THs is perhaps an indication of the effectiveness of used plant species. This is attributed to the fact that these healers are known to combine species for the increased efficacy [88]. Use of single therapies by Bapedi THs might also be due to simplifying the preparation and because of the nature of asthma. For instance asthma attack is in most cases sudden and thus requires immediate medical attention. In light of this an overwhelming majority of THs in this study might prefer preparing medicine from a single species (which is both straightforward and less time consuming) in case of exigency. Only 24.8% (n=38) of the herbal preparations used by interviewed Bapedi THs were multitherapies (Table 1). Healers who utilised this recipe disclosed that it enhances the effectiveness of medicine, which could be due to synergistic effects of several plant compounds that are active singly. However, this postulation warrants further investigations.

Remedies were prepared via boiling, macerating, pounding, squeezing, and rubbing and raw (prescribed as harvested). Harvested parts from certain plant were prepared using more than one method or a same technique was used differently amid THs (Table 1). Boiling (48.3%, n=74), pounding (45%, n=69), pounding and boiling (2.6%, n=4), chewing and macerating (1.3%, n=2, for each), rubbing (0.6%, n=1), and squeezing and pounding (0.6%, n=1), respectively, were the principal methods of herbal preparation in the present study. Most of these methods are consistently reported in various ethnobotanical surveys conducted in Africa [17, 18] and elsewhere [89] focusing on asthma. High usage of boiling plant parts by Bapedi THs might be due to the simplicity of preparation. Bapedi THs prefer pounded remedies because they have a far longer shelf life for the preparation [90]. Depending on an individual healer's preference, a minimum of two to a maximum of 14 minutes was used to boil various plant parts. Plant parts were pounded with grinding stones and metal equipment. Preparation times of these parts via maceration technique by Bapedi THs took from three to 24 hours (depending on an individual healer), which could explain its limited preference in this study. On the other hand, limited utilisation of squeezing and rubbing (n=1, for each) amongst Bapedi THs might be attributed to the seasonal availability of fresh fruits and leaves, respectively (Table 1).

The present study further assessed the different modes of application of the prepared remedies. Accordingly, of the 153 recorded recipes used for asthma and related symptoms, 80.9% (n=123) were administered orally, 19% (n=29) nasally, and 0.6% (n=1) topically. Naoumi [17] also found that most of the asthma medicines in his study are administered orally with very few which were taken topically. Distinct preferences of oral as route of herbal administration by Bapedi THs might be attributed to its convenience, for instance, it is straightforward and thus requires no special training. In addition its dosages can easily be premeasured.

Dosage strength of herbal remedy recorded in this study was also determined (Table 1). Overall there was a high consistency with regard to the boiled medicines taken orally. For example, a metal cup (500 ml) full of liquid preparations was prescribed by all THs three times a day (morning, midday, and evening). However, dosage inconsistency amongst interviewed Bapedi THs was noted for some preparations. This included boiled medicines administered nasally under a blanket, the dosage strength of which depended on an individual healer. Similarly, depending on individual healer two to five table spoons of pounded plant parts were mainly prescribed with a metal cup (500 ml) full of warm water. Some THs prescribed pounded plant parts with this cup but full of Mageu® drink or soft porridge. Lack of precision and standardization in the measurement of herbal medicine amongst Bapedi THs is one weakness of their traditional healthcare system.

## 4. Conclusions

The present study is the first to explore plants used traditionally to treat asthma and related conditions in South Africa. Overall the most widely distributed and highly used medicinal plants by all interviewed Bapedi THs (n=140) who treated asthma were* C. ternatum*,* C. transvaalensis*,* L. caffer*,* E. axillare*,* M. obovata*,* S. birrea*, and* S. natalensis*. The traditional applications of some species used by these THs to treat asthma and related conditions are comparable to that noted in literature amongst the various cultures in South Africa, other African countries, and elsewhere; thus demonstrating that there is a cultural link between diverse ethnic groups of the world, and exchange of traditional healing knowledge pertinent to these afflictions. Our study also recorded a larger number of new records of known medicinal plants used in traditional healing by various cultures across South Africa and Africa at large, a finding which contributes towards establishments of an African database of antiasthma plants and a new solid lead towards search for bioactive compounds against asthma.

## Figures and Tables

**Table 1:** Plant used to treat asthma and related symptoms in the Capricorn, Sekhukhune, and Waterberg districts of Limpopo Province, South Africa.

Botanical family	**Species names**	**Vernacular name**	**Habit**	**Used plant parts**	**State of use**	**Methods of herbal ** **preparation and administration**	**Aliment/s treated**	**Frequency** ** of use;** **n=THs ** **(140)**		**FL**	**UV**
								**UM**	**%**		
Acanthaceae	*Blepharis diversispina* (Nees) C. B. Clarke	Setlwatlwa	Shrub	Root	Dry	Boiled for 3–5 minutes. Extract is taken orally. Thrice a day	Fatigue	**6**	**4.2**	**100**	**0.04**

Acanthaceae	*Blepharis subvolubilis* C. B. Clarke	Mookapitsi	Shrub	Root	Dry	Boiled for 5–6 minutes. Extract is taken orally. Thrice a day	Fatigue	**1**	**0.7**	**100**	**0.00**

Alliaceae	*Tulbaghia violacea* Harv. var. violacea	Moeye-ya-naga	Herb	Bulb	Fresh	Boiled for 5 minutes. Steam inhaled (nasally) under blanket. Thrice a day	Nasal congestion	**2**	**1.4**	**100**	**0.01**

Amaryllidaceae	^**∗**^ *Allium sativum* L.	Khonofolo	Herb	Bulb	Dry	Pounded and taken orally with warm water. Thrice a day	Asthma	**1**	**0.7**	**50**	**0.01**
							Laboured breathing	**1**	**0.7**	**50**	

Amaryllidaceae	*Clivia caulescens* R. A. Dyer	Maime	Herb	Root	Dry	Boiled for 4–10 minutes. Extract is taken orally. Thrice a day	Asthma	**3**	**2.1**	**100**	**0.02**

Anacardiaceae	*Harpephyllum caffrum* Bernh. ex Krauss	Motšhidi-tshwene	Tree	Root	Dry	Boiled for 5 minutes. Extract is taken orally. Thrice a day	Asthma	**1**	**0.7**	**100**	**0.00**

Anacardiaceae	^**∗**^ *Mangifera indica* L	Momenko	Tree	Root	Fresh	Boiled for 5 minutes. Extract is taken orally. Thrice a day	Asthma	**1**	**0.7**	**100**	**0.00**

Anacardiaceae	^**∗**^ *Schinus molle* L.	Thoba/ Mokwepere	Tree	Leaf	Fresh	Boiled for 5–7 minutes. Steam is inhaled (nasally) under blanket. Thrice a day	Asthma	**1**	**0.7**	**14.2**	**0.05**
							Fatigue	**4**	**2.8**	**57.1**	
							Laboured breathing	**2**	**1.4**	**28.5**	

Anacardiaceae	*Sclerocarya birrea* (A.Rich.) Hochst. subsp. caffra (Sond.)	Morula/ Mokano	Tree	Bark	Dry	Pounded and mixed with dried powdered roots of *B. discolor* and *S. italica, *and dried bark of* P. africanum. *Powered is poured in to the boiled water and steam is inhaled (nasally) under blanket. Thrice a day	Asthma	**1**	**100**	**100**	**1**
						Pounded and mixed with dried powdered roots of *A. erioloba*, *X*. *caffra, *anddried bark of* P*. *africanum*. Taken orally with warm water	Asthma	**1**			
						Pounded and taken orally with warm water. Thrice a day	Asthma	**1**			
				Fruit	Fresh	Juice is squeezed (raw), dried. and pounded. Powder is poured in the hot water. Steam is inhaled (nasally) under blanket. Thrice a day	Asthma	**137**			

Apiaceae	*Alepidea amatymbica* Eckl. & Zeyh. var. amatymbica	Lešokwane	Herb	Rhizome	Dry	Pounded and taken orally with warm water. Thrice a day	Asthma	**11**	**7.8**	**100**	**0.07**

Apocynaceae	*Schizoglossum nitidum* Schltr	Phenyokga	Herb	Root	Dry	Boiled (until water gains colour) and extract is taken orally. Thrice a day	Asthma	**2**	**1.4**	**66.6**	**0.02**
							Nasal congestion	**1**	**0.7**	**33.3**	

Apocynaceae	*Strophanthus speciosus* (Ward & Harv.) Reber	Morarwane	Shrub	Root	Dry	Boiled for 6–12 minutes. Extract is taken orally. Thrice a day	Asthma	**15**	**10.7**	**100**	**0.10**

Araceae	^**∗**^ *Stylochaeton natalensis* Schott	Mokunya/ Mokušhete	Herb	Root	Dry	Boiled for 5 minutes. Extract is taken orally. Thrice a day	Asthma	**140**	**100**	**100**	**1**

Araceae	*Zantedeschia aethiopica * (L.) Spreng.	Mothebe	Herb	Root	Dry	Boiled for 5–6 minutes. Extract is taken orally. Thrice a day	Asthma	**7**	**5**	**100**	**0.05**

Asphodelaceae	*Aloe *spp.	Thogo/ Marobadibogale	Shrub	Leaf	Fresh	Mixed with dried pounded leaf of* D. senecioides. *Boiled for 2 minutes. Extract is taken orally. Thrice a day	Asthma	**3**	**2.1**	**37.5**	**0.02**
						Macerated in warm for 3–24 hrs. Decoction is taken orally. Thrice a day	Fatigue	**5**	**3.5**	**62.5**	**0.03**

Asphodeloideae	*Aloe falcata* Baker	Sekgopha	Shrub	Leaf	Fresh	Macerated in warm water 2–5 hrs. Decoction is taken orally. Thrice a day	Fatigue	**1**	**0.7**	**100**	**0.00**

Asteraceae	*Artemisia afra* Jacq. ex Willd. var. afra	Legana/ Moilanši	Herb	Leaf	Dry	Boiled for 3–7 minutes. Extract is taken orally. Thrice a day	Wheezing	**2**	**23.5**	**6**	**0.23**
						Mixed with fresh leaf of *C. sativa*. Boiled for 5 minutes. Extract is taken orally. Thrice a day	Asthma	**8**	**17.8**	**75.7**	
						Pounded and mixed with dried powered bark of *P. africanum*. Taken orally with Syrup®. Thrice a day	Asthma	**1**			
						Boiled for 4–5 minutes. Steam is inhaled (nasally) under blanket. Thrice a day	Asthma	**16**			
							Nasal congestion	**6**	**4.2**	**18.1**	

Asteraceae	*Callilepis laureola* DC.	Phela/Hlonya/ Makuru/Pedipekanto	Herb	Root	Dry	Boiled for 4–6 minutes. Extract is taken orally. Thrice a day	Asthma	**10**	**9.2**	**86.6**	**0.10**
						Pounded and mixed with dried powdered root of* Z. capense*. Taken orally with warm water. Thrice a day	Asthma	**1**			
						Mixed with fresh leaf of *C. edulis*. Boiled for 5 minutes. Extract is taken orally. Thrice a day	Asthma	**1**			
						Pounded and mixed with dried powered root of *L. javanica*. Boiled for 4 minutes. Steam is inhaled (nasally) under blanket. Thrice a day	Asthma	**1**			
						Boiled for 7–9 minutes. Extract is taken orally. Thrice a day	Fatigue	**1**	**1.4**	**13.3**	
						Pounded and mixed with dried powered whole plants of* H. caespititium,* leaves of* L. javanica *and* T. dioica*, and root of *O. lanceolata*. Taken orally with warm water. Thrice a day	Fatigue	**1**			

Asteraceae	*Dicoma anomala* subsp. gerrardii	Phelana/ Makušwaneng	Herb	Root	Dry	Boiled for 5–8 minutes. Extract is taken orally. Thrice a day Or Pounded and taken orally with warm water. Thrice a day	Asthma	**3**	**2.1**	**100**	**0.02**

Asteraceae	*Helichrysum caespititium* (DC.) Harv.	Bokgatha	Herb	Whole plant	Dry	Pounded and mixed with dried powdered roots of *C. laureola* and *O. lanceolata, *leaves of* L. javanica *and* T. dioica*. Taken orally with warm water. Thrice a day	Fatigue	**1**	**0.7**	**100**	**0.00**

Asteraceae	*Helichrysum gymnocomum* DC.	Mpepho	Herb	Whole plant	Dry	Pounded and taken orally with warm water. Thrice a day	Asthma	**2**	**1.4**	**100**	**0.01**

Asteraceae	^**∗**^ *Pseudognaphalium luteo-* *album* (L.) Hilliard & B.L.Burtt	Unknown	Herb	Leaf	Dry	Boiled for 5 minutes. Extract is taken orally. Thrice a day	Fatigue	**2**	**1.4**	**100**	**0.01**

Asteraceae	*Psiadia punctulata* (DC.) Vatke	Lesotlane/ Monotletšane/lesodi	Shrub	Root	Dry	Pounded and taken orally with warm water. Thrice a day	Asthma	**7**	**5**	**53.8**	**0.05**
						Boiled for 5–7 minutes. Steam is inhaled under (nasally) blanket. Thrice a day	Nasal congestion	**6**	**4.2**	**46.1**	**0.04**

Asteraceae	^**∗**^ *Schkuhria pinnata* (Lam.) Kuntze ex Thell.	Šathume/ Mošašane/Seralane	Herb	Whole plant	Fresh	Pounded and mixed with dried powdered root of *P. grandiflora.* Powder is poured in the boiled water. Steam inhaled (nasally) under blanket. Thrice a day	Asthma	**1**	**0.7**	**100**	**0.00**

Asteraceae	*Senecio serratuloides* DC.	Legatuludi	Shrub	Leaf	Dry	Boiled for 5 minutes. Steam is inhaled (nasally) under blanket. Thrice a day	Asthma	**4**	**2.8**	**33.3**	**0.18**
						Pounded and taken orally with warm water. Thrice a day	Fatigue	**4**	**2.8**	**33.3**	
							Wheezing	**4**	**2.8**	**33.3**	

Asteraceae	*Vernonia natalensis* Sch.Bip. e x Walp.	Mošuhla	Herb	Leaf	Leaf	Pounded and taken orally with warm water. Thrice a day	Asthma	**14**	**19.2**	**51.8**	**0.19**
						Boiled for 3–8 minutes and steam is inhaled (nasally) under blanket. Thrice a day	Nasal congestion	**12**	**9.2**	**48.1**	
						Boiled for 5–13 minutes. Extract is taken orally. Thrice a day	Nasal congestion	**1**			

Cactaceae	^**∗**^ *Opuntia ficus-indica* (L.) Mill.	Motloro	Tree	Root	Dry	Pounded and taken orally with warm water. Thrice a day	Asthma	**1**	**0.7**	**100**	**0.00**

Canellaceae	*Warburgia salutaris* (G.Bertol.) Chiov.	Molaka	Tree	Bark	Dry	Boiled for 4–11 minutes. Extract is taken orally. Thrice a day	Asthma	**4**	**2.8**	**36.3**	**0.07**
							Wheezing	**7**	**5**	**63.6**	

Cannabaceae	*Cannabis sativa* L. var. indica (Lam.) Wehmer	Lebake/Patše	Herb	Leaf	Fresh	Mixed with dried leaf of *A. afra*. Boiled for 5 minutes. Extract is taken orally. Thrice a day	Asthma	**8**	**5.7**	**42.1**	**0.13**
					Dry	Pounded and taken orally with warm water. Thrice a day	Tight chest	**11**	**7.8**	**57.8**	

Capparaceae	*Maerua juncea* Pax subsp. crustata (Wild) Wild	Diragadibonwe	Shrub	Root	Dry	Boiled for 5 minutes. Extract is taken orally. Thrice a day	Asthma	**3**	**2.1**	**50**	**0.02**
						Mixed with fresh bulb of* D*. *elata*, dried bark of *C. abbreviata*. Boiled for 6 minutes. Extract is taken orally. Thrice a day	Fatigue	**1**	**2.1**	**50**	**0.02**
						Pounded and taken orally with warm water. Thrice a day	Fatigue	**2**			

Celastraceae	*Catha edulis* (Vahl) Forssk. ex Endl.	Lehlatse/Lewang/ Molomomonate	Tree	Root	Dry	Boiled for 5 minutes. Extract is taken orally. Thrice a day	Fatigue	**1**	**0.7**	**100**	**0.00**

Convolvulaceae	*Ipomoea albivenia* (Lindl.) Sweet	Mošope	Shrub	Root	Dry	Boiled for 4–5 minutes. Extract is taken orally. Thrice a day	Asthma	**1**	**0.7**	**100**	**0.00**

Crassulaceae	*Kalanchoe brachyloba* Welw. ex Britten	Moethi/ Mošimanewanaga/ Moritšikana	Shrub	Leaf	Fresh	Rubbed (raw) between hands and vapour is inhaled (nasally). Thrice a day	Nasal congestion	**13**	**9.2**	**100**	**0.09**

Cucurbitaceae	*Cucumis metuliferus* E.Mey. ex Naudin	Tšhitšhi	Herb	Root	Dry	Pounded and taken orally with warm water. Thrice a day	Asthma	**4**	**2.8**	**100**	**0.02**

Cyperaceae	*Cyperus sexangularis*Nees	Mohlahla	Herb	Root	Dry	Pounded and taken orally with warm water. Thrice a day	Asthma	**7**	**5**	**10.7**	**0.46**
							Fatigue	**58**	**41.4**	**89.2**	

Dioscoreaceae	*Dioscorea sylvatica* Eckl. var. brevipes (Burtt Davy) Burkill	Kgato	Herb	Tuber	Dry	Pounded and taken orally with warm water. Thrice a day	Asthma	**10**	**7.1**	**100**	**0.07**

Euphorbiaceae	*Croton gratissimus* Burch. var. *gratissimus*	Moolologa/ Selogane	Tree	Root	Dry	Boiled for 6–10 minutes. Extract is taken orally. Thrice a day	Asthma	**12**	**8.5**	**48**	**0.17**
							Wheezing	**8**	**5.7**	**32**	
						Or					
						Pounded and taken orally with warm water. Thrice a day					
						Boiled for 9 minutes. Steam is inhaled (nasally) under blanket. Thrice a day	Nasal congestion	**5**	**3.5**	**20**	

Euphorbiaceae	*Euphorbia schinzii* Pax	Ngaka-dianya	Herb	Root	Dry	Boiled for 5–8 minutes. Steam is inhaled (nasally) under blanket. Thrice a day	Asthma	**19**	**13.5**	**100**	**0.13**

Euphorbiaceae	*Jatropha zeyheri* Sond.	Sephapabadiya	Herb	Root	Dry	Boiled for 5 minutes. Extract is taken orally. Thrice a day	Asthma	**1**	**0.7**	**100**	**0.00**

Euphorbiaceae	*Tragia dioica* Sond.	Bogopa/ Mabatšane	Herb	Leaf	Dry	Pounded and mixed with dried powered whole plant of* H. caespititium,* leaf of* L. javanica,* and roots of* O. lanceolata* and *C. laureola*. Taken orally with warm water. Thrice a day	Fatigue	**1**	**0.7**	**100**	**0.00**

Fabaceae	*Acacia erioloba* E.Mey.	Mogohlo/ Mošu	Tree	Root	Dry	Pounded and mixed with dried powdered bark of* P. africanum* and *S*. *birrea* and root of *X*. *caffra*. Taken orally with warm water. Thrice a day	Asthma	**1**	**0.7**	**100**	**0.00**

Fabaceae	*Acacia sieberiana* DC. var. woodii (Burtt Davy) Keay & Brenan	Mošu	Tree	Root	Dry	Pounded and taken orally with warm water. Thrice a day	Asthma	**1**	**0.7**	**100**	**0.00**

Fabaceae	*Acacia senegal* (L.) Willd. var. rostrata Brenan	Mokgaripe	Tree	Root	Dry	Boiled for 6–11 minutes. Extract is taken orally. Thrice a day	Asthma	**2**	**1.4**	**100**	**0.01**

Fabaceae	*Albizia adianthifolia* (Schumach.) W.Wight var. Adianthifolia	Mafahla-nare	Tree	Root	Dry	Boiled for 5–12 minutes. Extract is taken orally. Thrice a day Or Pounded and taken orally with warm water. Thrice a day	Fatigue	**3**	**2.1**	**100**	**0.02**

Fabaceae	*Cassia abbreviata* Oliv. subsp. beareana (Holmes) Brenan	Monepenepe	Tree	Bark	Dry	Boiled for 5–9 minutes. Extract is taken orally. Thrice a day	Asthma	**3**	**2.1**	**100**	**0.02**

Fabaceae	*Elephantorrhiza burkei* Benth.	Mošišane/ Batswetsi	Shrub	Root	Dry	Pounded and mixed with dried powdered stem of* A. spinosa* and root of* P. africanum. *Taken orally with warm water. Thrice a day	Fatigue	**1**	**2.1**	**50**	**0.04**
						Boiled for 5–8 minutes. Extract is taken orally. Thrice a day	Fatigue	**2**			
							Asthma	**3**	**2.1**	**50**	

Fabaceae	*Elephantorrhiza goetzei* (Harms) Harms subsp. Goetzei	Mošitšane	Shrub	Root	Dry	Mixed with fresh bulb of* E. autumnalis*. Boiled for 6 minutes. Extract is taken orally. Thrice a day	Fatigue	**1**	**0.7**	**100**	**0.00**

Fabaceae	*Erythrina lysistemon* Hutch.	Sebalo/ Mmale	Tree	Bark	Dry	Boiled for 5–8 minutes. Extract is taken orally. Thrice a day	Wheezing	**10**	**7.1**	**100**	**0.07**

Fabaceae	*Peltophorum africanum* Sond.	Mosehla	Tree	Bark	Dry	Pounded and mixed with dried powered leaf of *A*. *afra*. Taken orally with Syrup®. Thrice a day	Asthma	**1**	**2.1**	**21.4**	**0.1**
						Pounded and mixed with a dried powdered root of *B. discolor* and *S. italica *and dried bark of* S. birrea. *Powder is poured in to the boiled water and steam is inhaled (nasally) under blanket. Thrice a day	Asthma	**1**			
						Pounded and mixed with dried powdered roots of *A. erioloba*, *X*. *caffra,* and dried bark of* S*. *birrea*. Taken orally with warm water. Thrice a day	Asthma	**1**			
						Boiled for 5–11 minutes. Extracts taken orally. Thrice a day	Fatigue	**10**	**7.8**	**78.5**	
				Root		Pounded and mixed with dried powdered stem of* A. spinosa* and a root of* E. burkei. *Taken orally with warm water. Thrice a day	Fatigue	**1**			

Fabaceae	*Senna italica* Mill. subsp. arachoides (Burch.) Lock	Moroteladitšhoši	Herb	Root	Dry	Pounded and mixed with dried powdered roots of *B. discolor, *dried bark of S. *birrea* and* P. africanum. * Powder is poured in to the boiled water and steam is inhaled (nasally) under blanket. Thrice a day	Asthma	**1**	**6.4**	**100**	**0.06**
						Boiled for 4–8 minutes. Extract is taken orally. Thrice a day	Asthma	**8**			

Gentianaceae	*Enicostema axillare* (Lam.) A. Raynal subsp. Axillare	Makgonotšohle/ Mphedu-ya-thaba	Herb	Whole plant	Dry	Boiled for 5–14 minutes. Extract is taken orally. Thrice a day. Or Pounded and taken orally with warm water. Thrice a day.	Asthma	**140**	**100**	**100**	**1**

Hyacinthaceae	*Drimia elata* Jacq.	Sekanama	Herb	Bulb	Fresh	Mixed with dried root of* M. juncea*, dried bark of *C. abbreviata*. Boiled for 6 minutes. Extract is taken orally. Thrice a day.	Fatigue	**1**	**0.7**	**8.3**	**0.08**
						Boiled for 6 minutes. Extract is taken orally. Thrice a day.	Fatigue	**3**	**2.1**	**25**	
							Wheezing	**4**	**2.8**	**33.3**	
						Boiled for 5 minutes. Steam is inhaled (nasally) under blanket. Thrice a day.	Asthma	**4**	**2.8**	**33.3**	

Hyacinthaceae	*Eucomis autumnalis* (Mill.) Chitt.	Mathubadifala	Herb	Bulb	Fresh	Boiled for 5 minutes. Extract is taken orally. Thrice a day.	Nasal congestion	**8**	**5.7**	**88.8**	**0.05**
						Mixed with dried root of* E. goetzei*. Boiled for 6 minutes. Extract is taken orally. Thrice a day.	Fatigue	**1**	**0.7**	**11.1**	**0.00**

Hyacinthaceae	*Eucomispallidiflora* Baker subsp. pole-evansii (N.E.Br.) Reyneke ex J.C.Manning	Mathubadifala	Herb	Bulb	Fresh	Boiled for 5–8 minutes. Steam is inhaled (nasally) under blanket. Thrice a day.	Nasal congestion	**11**	**7.8**	**52.2**	**0.15**
						Boiled for 5–10 minutes. Extract is taken orally. Thrice a day.	Fatigue	**9**	**6.4**	**42.8**	
							Wheezing	**1**	**0.7**	**4.7**	

Hypoxidaceae	*Hypoxis hemerocallidea* Fisch., C.A.Mey. & Avé- Lall	Hlakudiboya/Titikwane/ Sekgekolwana	Herb	Tuber	Fresh	Mixed with fresh bulb of* S. aethiopicus.* Boiled for 5 minutes. Extract is taken orally. Thrice a day.	Asthma	**1**	**10**	**77.7**	**0.12**
						Boiled for 5–12 minutes. Extract is taken orally. Thrice a day.	Asthma	**13**			
							Fatigue	**4**	**2.8**	**22.2**	

Hypoxidaceae	*Hypoxis obtusa* Burch. ex Ker Gawl.	Monna maledu	Herb	Tuber	Fresh	Boiled for 4–7 minutes. Extract is taken orally. Thrice a day.	Fatigue	**2**	**1.4**	**100**	**0.01**

Icacinaceae	*Cassinopsis ilicifolia* (Hochst.) Kuntze	Mohufi/Mohufe	Tree	Root	Dry	Boiled for 5–7 minutes. Extract is taken orally. Thrice a day.	Fatigue	**2**	**1.4**	**100**	**0.01**

Icacinaceae	*Pyrenacantha grandiflora* Baill.	Bjere	Shrub	Root	Dry	Pounded and mixed with dried powdered entire plant of *S. pinnata.* Powder is poured in the boiled water. Steam inhaled (nasally) under blanket. Thrice a day.	Asthma	**1**	**0.7**	**100**	**0.01**

Kirkiaceae	*Kirkia wilmsii* Engl.	Modumela/ mogaba	Tree	Bark	Fresh	Boiled for minutes. Steam is inhaled (nasally) under blanket. Thrice a day.	Asthma	**2**	**1.4**	**66.6**	**0.02**
							Nasal congestion	**1**	**0.7**	**33.3**	

Lamiaceae	*Clerodendrum glabrum* E.Mey. var. angustifolium E.Mey.	Mohlokohloko	Tree	Leaf	Fresh	Boiled for 5–8 minutes. Extract is taken orally. Thrice a day.	Asthma	**4**	**2.8**	**100**	**0.02**

Lamiaceae	*Clerodendrum ternatum * Schinz	Sebokane	Herb	Whole plant	Dry	Pounded and taken orally with warm water. Thrice a day.	Asthma	**140**	**100**	**100**	**1**

Lamiaceae	*Leonotis leonurus* (L.) R.Br.	Lebake	Shrub	Root or leaf	Dry	Pounded and taken orally with warm water. Thrice a day.	Asthma	**14**	**10**	**100**	**0.1**

Lauraceae	*Cryptocarya transvaalensis* Burtt Davy	Kgosupsa	Tree	Bark	Dry	Boiled for 5–9 minutes. Extract is taken orally. Thrice a day.	Asthma	**140**	**100**	**100**	**1**

Malvaceae	*Abutilon galpinii* A. Meeuse	Mmotša	Shrub	Root	Dry	Boiled for 5–8 minutes. Extract is taken orally. Thrice a day.	Asthma	**5**	**3.5**	**100**	**0.03**

Malvaceae	*Adansonia digitata*	Mogoo	Tree	Root	Dry	Boiled for 6–10 minutes. Extract is taken orally. Thrice a day.	Fatigue	**68**	**48.5**	**100**	**0.48**

Malvaceae	*Dombeya rotundifolia* (Hochst.) Planch. var. rotundifolia	Mokgoba	Tree	Root	Dry	Pounded and extract is taken orally with warm water. Thrice a day.	Fatigue	**3**	**2.1**	**100**	**0.02**

Malvaceae	*Gossypium herbaceum* L. subsp. africanum (Watt) Vollesen	Katluni/Leokodi/ Mohlare-wa-mawisi	Shrub	Root	Dry	Pounded and extract is taken orally with warm water. Thrice a day.	Asthma	**70**	**50**	**100**	**0.5**

Malvaceae	*Grewia hispida* Harv.	Mogwete/ Mogolori/Lefielo	Shrub	Root	Dry	Pounded and extract is taken orally. Thrice a day.	Nasal congestion	**2**	**1,4**	**100**	**0.01**

Malvaceae	*Grewia sulcata* Mast. var. sulcata	Mogwete/Mogoto	Tree	Root	Dry	Pounded and taken orally with warm water. Thrice a day.	Asthma	**2**	**1.4**	**100**	**0.01**

Malvaceae	*Sida cordifolia* L.	Mohutasela	Shrub	Root	Dry	Pounded and taken orally with warm water. Thrice a day.	Asthma	**3**	**2.1**	**100**	**0.02**

Mesembryanthemaceae	*Carpobrotus edulis* (L.) L. Bolus subsp. edulis	Mošhipse	Herb	Leaf	Fresh	Chewed (orally) as raw and juice is swallowed. Thrice a day	Asthma	**3**	**2.8**	**100**	**0.02**
						Mixed with dried root of *C*. *laureola*. Boiled for 5 minutes. Extract is taken orally. Thrice a day.	Asthma	**1**			

Moringaceae	^**∗**^ *Moringa oleifera* sensu Exell & Mendon	Moringka	Tree	Leaf	Dry	Pounded and taken orally with warm water. Thrice a day	Fatigue	**17**	**12.1**	**100**	**0.12**

Myrtaceae	^**∗**^ *Eucalyptus camaldulensis * *Dehnh*	Mopilikomo	Tree	Bark	Dry	Boiled for 5 minutes. Steam is inhaled (nasally) under blanket. Thrice a day	Asthma	**1**	**0.7**	**100**	**0.00**

Olacaceae	*Ximenia caffra* Sond. var. natalensis Sond.	Motšhidi-kgomo	Tree	Root	Dry	Pounded and mixed with dried powdered roots of *A. A. erioloba*, dried bark of* P*. *africanum *and* S. birrea*. Taken orally with warm water. Thrice a day	Asthma	**1**	**5.7**	**100**	**0.05**
						Pounded and taken orally with warm water. Thrice a day	Asthma	**7**			

Oleaceae	*Olea europaea* L. subsp. africana (Mill.) P. S. Green	Mohlware/ Mo-olive	Tree	Root	Dry	Pounded and taken orally with warm water. Thrice a day	Asthma	**4**	**3.5**	**100**	**0.03**
						Boiled for 8 minutes and extract is taken orally. Thrice a day	Asthma	**1**			

Punicaceae	^**∗**^ *Panica granatum* L.	Mokgarenate	Tree	Fruit scale	Fresh	Chew as raw (orally). Thrice a day	Asthma	**1**	**0.7**	**100**	**0.00**

Passifloraceae	*Adenia fruticosa* Burtt Davy subsp. fruticosa	Mopowane	Shrub	Root	Dry	Boiled for 5–13 minutes. Extract is taken orally. Thrice a day	Asthma	**9**	**9.2**	**69.2**	**0.09**
						Boiled for 6 minutes. Extract is used topically as bath. Thrice a day	Fatigue	**4**	**2.8**	**30.7**	

Passifloraceae	*Adenia spinosa* Burtt Davy	Monna-apare/ Pisayabatšumi/ Mothema	Shrub	Stem	Dry	Pounded and taken orally with warm water. Thrice a day	Asthma	**25**	**17.8**	**30.4**	**0.58**
							Fatigue	**56**	**40.7**	**69.5**	
						Pounded and mixed with dried powdered roots of* E. burkei* and* P. africanum. *Taken orally with warm water. Thrice a day	Fatigue	**1**			

Pedaliaceae	*Dicerocaryum senecioides * (Klotzsch) Abels	Momphati	Herb	Leaf	Dry	Pounded and mixed with fresh leaf of* Aloe spp.*Boiled for 2 minutes. Extract is taken orally. Thrice a day	Asthma	**3**	**2.1**	**100**	**0.02**

Plumbaginaceae	*Plumbago zeylanica* L.	Mašimabe/ Mašegomabe	Shrub	Root	Dry	Boiled for 6–13 minutes. Extract is taken orally. Thrice a day	Asthma	**4**	**2.8**	**80**	**0.03**
							Nasal congestion	**1**	**0.7**	**20**	

Poaceae	*Sorghum bicolor* (L.) Moench subsp. arundinaceum (Desv.) de Wet & Harlan	Mabele-thoro	Herb	Seed	Dry	Pounded and taken orally with Mageu® drink or soft porridge. Thrice a day	Fatigue	**6**	**4.2**	**100**	**0.04**

Poaceae	^**∗**^ *Zea mays* subsp. mays L.	Mabele	Herb	Root	Dry	Pounded and taken orally with warm water. Thrice a day	Asthma	**1**	**0.7**	**100**	**0.00**

Polygalaceae	*Securidaca * *longepedunculata* Fresen. var. longepedunculata	Mphesu/ Mpitlamarago	Tree	Root	Dry	Boiled for 5–10 minutes. Steam is inhaled (nasally) under blanket. Thrice a day	Asthma	**2**	**1.4**	**100**	**0.01**

Proteaceae	*Protea caffra* Meisn. subsp. caffra	Modumela	Tree	Root	Dry	Pounded and taken orally with warm water. Thrice a day	Asthma	**8**	**5.7**	**88.8**	**0.06**
							Fatigue	**1**	**0.7**	**11.1**	

Ptaeroxylaceae	*Ptaeroxylon obliquum* (Thunb.) Radlk.	Mogabaletswana	Tree	Root	Dry	Pounded and taken orally with warm water. Thrice a day	Asthma	**25**	**17.8**	**100**	**0.17**

Rhamnaceae	*Berchemia discolor* (Klotzsch) Hemsl.	Moneyee/ Mogokgoma	Tree	Root	Dry	Pounded and mixed with powdered dried bark of *S. birrea* and *P. africanum* and dried root of *S. italica. *Powder is poured in boiled water and steam is inhaled (nasally) under blanket. Thrice a day	Asthma	**1**	**0.7**	**100**	**0.00**

Rhamnaceae	*Ziziphus mucronata* Willd. subsp. mucronata	Mokgalo	Tree	Root	Dry	Pounded and taken orally with warm water. Thrice a day	Asthma	**1**	**0.7**	**100**	**0.00**

Rutaceae	^**∗**^ *Citrus limon* (L.) Burm.f.	Moswiri	Tree	Fruit	Fresh	Boiled for 4-5 minutes. Extract is taken orally. Thrice a day	Tight chest	**2**	**1.4**	**100**	**0.01**

Rutaceae	Vepris *reflexa* I. Verd.	Pharagobe	Tree	Root	Dry	Pounded and taken orally with warm water. Thrice a day	Fatigue	**2**	**1.4**	**100**	**0.01**

Rutaceae	*Zanthoxylum capense* (Thunb.) Harv.	Monokwane/ Moregakgaka	Tree	Root	Dry	Pounded and taken orally with warm water. Thrice a day	Wheezing	**7**	**5**	**53.8**	**0.09**
							Asthma	**5**	**3.5**	**46.1**	
						Pounded and mixed with dried powdered root of* C. laureola*. Taken orally with warm water. Thrice a day	Asthma	**1**	**0.7**		

Santalaceae	*Osyris lanceolata* Hochst. & Steud.	Mphera	Tree	Root	Dry	Pounded and taken orally with warm water. Thrice a day	Asthma	**14**	**11.4**	**87.5**	**0.11**
						Pounded and mixed with dried powdered root of *W. somnifera*. Taken orally with warm water. Thrice a day	Laboured breathing	**1**	**0.7**	**6.2**	
						Pounded and mixed with dried powdered root of *C. laureola* and* O. lanceolata*, whole plant of* H. caespititium, *and leaves of* L. javanica *and* T. dioica*. Taken orally with warm water. Thrice a day	Fatigue	**1**	**0.7**	**6.2**	

Sapindaceae	*Dodonaea viscosa* Jacq. var. angustifolia (L.f.) Benth.	Mofentshe	Tree	Root	Dry	Pounded and taken orally with warm water. Thrice a day	Laboured breathing	**1**	**0.7**	**100**	**0.00**

Sapotaceae	*Englerophytum * *magalismontanum* (Sond.) T. D. Penn.	Mohlatshwa	Tree	Root	Dry	Boiled for 5 minutes. Steam is inhaled (nasally) under blanket. Thrice a day	Asthma	**3**	**2.1**	**100**	**0.02**

Sapotaceae	*Mimusops obovata* Nees ex Sond.	Mmupudu	Tree	Root	Dry	Mixed with (spider's web). Pounded and taken orally with warm water. Thrice a day	Asthma	**140**	**100**	**100**	**1**

Scrophulariaceae	*Buddleja salvifolia *(L.) Lam	Moketla	Shrub	Root	Dry	Boiled for 5–10 minutes. Steam is inhaled (nasally) under blanket. Thrice a day	Asthma	**3**	**2.1**	**100**	**0.02**

Solanaceae	*Solanum catombelense* Peyr.	Mothola-o- momokhwibidu	Herb	Whole plant	Dry	Pounded and taken orally with warm water. Thrice a day	Asthma	**2**	**1.4**	**100**	**0.01**

Solanaceae	*Solanum panduriforme* E.Mey.	Mothola-o- moserolwane	Herb	Root	Dry	Pounded and taken orally with warm water. Thrice a day	Asthma	**3**	**2.1**	**100**	**0.02**

Solanaceae	*Withania somnifera* (L.) Dunal	Mošalašupeng	Shrub	Root	Dry	Pounded and taken orally with warm water. Thrice a day	Fatigue	**6**	**4.2**	**85.7**	**0.04**
						Pounded and mixed with dried powdered root of *O. lanceolata*. Taken orally with warm water. Thrice a day	Laboured breathing	**1**	**0.7**	**14.2**	**0.00**

Thymelaeaceae	*Lasiosiphon caffer* Meisn.	Nkekologe	Shrub	Root	Dry	Pounded and taken orally with warm water. Thrice a day	Asthma	**140**	**100**	**100**	**1.12**
							Tight chest	**17**	**12.1**	**10.8**	

Velloziaceae	*Xerophyta retinervis* Baker	Thuse	Herb	Root	Dry	Pounded and taken orally with warm water. Thrice a day	Asthma	**4**	**2.8**	**100**	**0.02**

Verbenaceae	*Lantana rugosa* Thunb.	Bokokotane/ mokokotane	Shrub	Leaf	Fresh	Boiled for 5–10 minutes. Steam is inhaled (nasally) under blanket. Thrice a day	Asthma	**5**	**3.5**	**100**	**0.03**

Verbenaceae	*Lippia javanica* (Burm.f.) Spreng	Mošunkwane/ motlaba-dipoo	Shrub	Leaf	Fresh	Pounded and mixed with dried powdered root of *C. laureola*. Boiled for 4 minutes and steam is inhaled (nasally) under blanket. Thrice a day	Asthma	**1**	**7.1**	**55.5**	**0.12**
						Boiled for 5–13 minutes. Steam is inhaled (nasally) under blanket. Thrice a day	Asthma	**9**			
					Dry	Pounded and mixed with dried powdered roots of *C. laureola* and* O. lanceolata*, whole plant of* H. caespititium,* and leaf of* T. dioica*. Taken orally with warm water. Thrice a day	Fatigue	**1**	**0.7**	**5.5**	
					Fresh	Boiled for 5–14 minutes. Extract is taken orally. Thrice a day	Laboured breathing	**2**	**1.4**	**11.1**	
						Boiled for 5–10 minutes. Steam is inhaled (nasally) under blanket. Thrice a day	Nasal congestion	**1**	**0.7**	**5.5**	
						Boiled for 5 minutes. Steam is inhaled (nasally) under blanket, but while eyes open. Thrice a day	Wheezing	**4**	**2.8**	**22.2**	

Vitaceae	*Rhoicissus tomentosa* (Lam.) Wild & R. B. Drumm.	Terebe-ya- nageng	Herb	Root	Dry	Pounded and taken orally with warm water. Thrice a day	Asthma	**4**	**2.8**	**100**	**0.02**

Zingiberaceae	*Siphonochilus aethiopicus* (Schweinf.) B. L. Burtt	Serokolo	Herb	Bulb	Fresh	Mixed with fresh bulb of *H*. *hemerocallidea*. Boiled for 5 minutes. Extract is taken orally. Thrice a day	Asthma	**1**	**0.7**	**100**	**0.00**

Exotic plant species: asterisk (^**∗**^); fidelity level: FL; use mention: UM; and use value: UV.

**Table 2:** Use of species to treat asthma (AS) and related symptoms within the districts and municipalities.

**Species name**	**Districts and municipalities**																			**Sum of overall ailment treated per species**
	**Capricorn**						**Sekhukhune**						**Waterberg**							
	**Aganang**	**Blouberg**	**Lepelle-** **Nkumpi**	**Molemole**	**Polokwane**	**Sum of ailments (FC)**	**Elias ** **Motsoaledi**	**Ephrime ** **Mogale**	**Fetakgomo**	**Makhudumathamaga**	**Tubatse**	**Sum of ** **ailments ** **(FC)**	**Bela-Bela**	**Lephalale**	**Modimolle**	**Mogalakwena**	**Mookgophong**	**Thabazimbi**	**Sum of ** **ailment (FC)**	
*Abutilon galpinii*	-	-	-	-	-	**0**	-	-	-	AS:5	-	**5**	-	-	-	-	-	-	**0**	**5**
*Acacia erioloba*	AS:1”	-	-	-	-	**1**	-	-	-	-	-	**0**	-	-	-	-	-	-	**0**	**1**
*Acacia senegal*	-	-	-	-	-	**0**	-	-	-	-	-	**0**	-	AS:2	-	-	-	-	**2**	**2**
*Acacia sieberiana*	AS:1	-	-	-	-	**1**	-	-	-	-	-	**0**	-	-	-	-	-	-	**0**	**1**
*Allium sativum*	AS:1	-	-	-	-	**1**	-	-	-	-	-	**0**	-	-	-	-	-	-	**0**	**1**
	LB:1	-	-	-	-	**1**	-	-	-	-	-	**0**	-	-	-	-	-	-	**0**	**1**
*Adansonia digitata*	FA: 14	FA: 15	FA:13	FA: 11	FA: 15	**68**	-	-	-	-	-	**0**	-	-	-	-	-	-	**0**	**68**
*Adenia fruticosa*	-	AS:2	AS:6	-	AS:1	**9**	-	-	-		-	**0**	-	-	-	-	-	-	**0**	**9**
	-	-	FA:4	-	-	**4**	-	-	-	-	-	**0**	-	-	-	-	-	-	**0**	**4**
*Adenia spinosa*	AS:1	-	-	AS:13	-	**14**	-	-	-	-	AS:4	**4**	-	AS:1	-	-	-	AS:6	**7**	**25**
	-	FA:1”:8	FA:1	-	-	**10**	FA:13	FA:10	FA:6	FA:6	FA:4	**39**	-	-	-	FA:8	-	-	**8**	**57**
*Albizia adianthifolia*	FA: 3	-	-	-	-	**3**	-	-	-	-	-	**0**	-	-	-	-	-	-	**0**	**3**
*Alepidea amatymbica*	AS:1	AS:1	-	AS:3	-	**5**	-	-	-	-	-	**0**	-	-	-	-	-	AS:6	**6**	**11**
*Aloe spp.*	-	-	-	-	-	**0**	-	-	-	-	-	**0**	-	-	-	-	AS:3”	-	**3**	**3**
	-	-	-	-	-	**0**	-	-	-	-	-	**0**	-	FA:5	-	-	-	-	**5**	**5**
*Aloe falcata*	-	-	-	-	-	**0**	-	-	-	-	-	**0**	-	-	-	-	-	FA:1	**1**	**1**
*Artemisia afra*	AS:4”	AS:3”	AS:1”	-	AS:7	**15**	-	-	AS:1”	AS:6	-	**7**	AS:3	-	-	-	-	-	**3**	**25**
	-	-	NC:6	-	-	**6**	-	-	-	-	-	**0**	-	-	-	-	-	-	**0**	**6**
	WH:2	-	-	-	-	**2**	-	-	-	-	-	**0**	-	-	-	-	-	-	**0**	**2**
*Berchemia discolor*	-	-	-	-	-	**0**	-	AS:1”	-	-		**1**	-	-	-	-	-	-	**0**	**1**
*Blepharis diversispina*	-	-	-	-	-	**0**	FA: 6	-	-	-	-	**6**	-	-	-	-	-	-	**0**	**6**
*Blepharis subvolubilis*	-	-	-	-	FA:1	**1**	-	-	-	-	-	**0**	-	-	-	-	-	-	**0**	**1**
*Buddleja salvifolia*	-	-	-	-	AS:3	**3**	-	-	-	-	-	**0**	-	-	-	-	-	-	**0**	**3**
*Callilepis laureola*	AS:1	-	-	-	AS:1”	**2**	-	AS:1”	-	-	AS:2	**3**	AS:2	AS:1	-	-	AS:1”:4	-	**8**	**13**
	FA:1	-	-	-	-	**1**	-	FA:1”	-	-	-	**1**	-	-	-	-	-	-	**0**	**2**
*Cannabis sativa*	AS:4”	AS:3”	-	-	-	**7**	-	-	AS:1”	-	-	**1**	-	-	-	-	-	-	**0**	**8**
	-	-	TC:11	-	-	**11**	-	-	-	-	-	**0**	-	-	-	-	-	-	**0**	**11**
*Carpobrotus edulis*	-	-	AS:1	-	AS:2	**3**	-	AS:1”	-	-	-	**1**	-	-	-	-	-	-	**0**	**4**
*Cassia abbreviata*	-	AS:3	-	-	-	**3**	-	-	-	-	-	**0**	-	-	-	-	-	-	**0**	**3**
	FA:1”:2	-	-	-	-	**3**	-	-	-	-	-	**0**	-	-	-	-	-	-	**0**	**3**
*Cassinopsis ilicifolia*	-	-	-	-	-	**0**	-	FA:2	-	-	-	**2**	-	-	-	-	-	-	**0**	**2**
*Catha edulis*	FA: 1	-	-	-	-	**1**	-	-	-	-	-	**0**	-	-	-	-	-	-	**0**	**1**
*Citrus limon*	-	-	TC:1	-	TC:1	**2**	-	-	-	-	-	**0**	-	-	-	-	-	-	**0**	**2**
*Clerodendrum glabrum*	-	-	-	-	-	**0**	-	AS:3	-	AS:1	-	**4**	-	-	-	-	-	-	**0**	**4**
*Clerodendrum ternatum*	AS:14	AS:15	AS:13	AS:11	AS:15	**68**	AS:13	AS:10	AS:6	AS:6	AS:4	**39**	AS:7	AS:5	-	AS:8	AS:7	AS:6	**33**	**140**
*Clivia caulescens *	-	-	-	AS: 3	-	**3**	-	-	-	-	-	0	-	-	-	-	-	-	0	3
*Croton gratissimus*	-	AS:3	-	AS:4	AS:5	**12**	-	-	-	-	-	**0**	-	-	-	-	-	-	**0**	**12**
	-	-	-	-	-	**0**	-	NC:1	NC:1	NC:2	NC:1	**5**	-	-	-	-	-	-	**0**	**5**
	-	-	-	-	-	**0**	-	-	-	-	-	**0**	WH:1	-	-	WH:3	WH:4	-	**8**	**8**
*Cryptocarya transvaalensis*	AS:14	AS:15	AS:13	AS:11	AS:15	**68**	AS:13	AS:10	AS:6	AS:6	AS:4	**39**	AS:7	AS:5	-	AS:8	AS:7	AS:6	**33**	**140**
*Cucumis metuliferus*	-	-	-	-	-	**0**	AS:1	-	-	AS:2	AS:1	**4**	-	-	-	-	-	-	**0**	**4**
*Cyperus sexangularis*	-	-	-	-	-	**0**	AS:1	-	AS:6	-	-	**7**	-	-	-	-	-	-	**0**	**7**
	FA:4	FA:7	-	-	FA:1	**12**	FA:13	FA:10	FA:6	FA:6	FA:4	**39**	FA:1	-	FA:3	-	FA:3	-	**7**	**58**
*Eucalyptus camaldulensis*	-	-	-	-	-	**0**	-	-	-	-	-	**0**	-	-	-	-	AS:1	-	**1**	**1**
*Dicerocaryum senecioides *	-	-	-	-	-	**0**	-	-	-	-	-	**0**	-	-	-	-	AS:3”	-	**3**	**3**
*Dicoma anomala*	-	-	-	-	-	**0**	-	-	-	-	-	**0**	-	AS:3	-	-	-	-	**3**	**3**
*Dioscorea sylvatica*	-	-	AS: 2	-	-	**2**	AS:1	-	-	AS:2	AS:1	**4**	-	AS:1	-	-	-	AS:3	**4**	**10**
*Dodonaea viscosa*	LB:1	-	-	-	-	**1**	-	-	-	-	-	**0**	-	-	-	-	-	-	**0**	**1**
*Dombeya rotundifolia*	-	-	-	-	FA:3	**3**	-	-	-	-		**0**	-	-	-	-	-	-	**0**	**3**
*Drimia elata*	-	-	-	-	-	**0**	AS:4	-	-	-	-	**4**	-	-	-	-	-	-	**0**	**4**
	FA:1”	-	-	-	FA:1	**2**	FA: 2	-	-	-	-	**2**	-	-	-	-	-	-	**0**	**4**
	-	WH: 4	-	-	-	**4**	-	-	-	-	-	**0**	-	-	-	-	-	-	**0**	**4**
*Elephantorrhiza burkei*	-	-	-	-	-	**0**	-	-	-	-	-	**0**	AS:3	-	-	-	-	-	**3**	**3**
	-	FA:1”:1	-	-	FA:1	**3**	-	-	-	-	-	**0**	-	-	-	-	-	-	**0**	**3**
*Elephantorrhiza goetzei*	FA:1”	-	-	-	-	**1**	-	-	-	-	-	**0**	-	-	-	-	-	-	**0**	**1**
*Englerophytum magalismontanum*	-	-	-	-	-	**0**	-	-	-	-	-	**0**	AS:1	-	-	AS:1	-	AS:1	**3**	**3**
*Enicostema axillare*	AS:14	AS:15	AS:13	AS:11	AS:15	**68**	AS:13	AS:10	AS:6	AS:6	AS:4	**39**	AS:7	AS:5	-	AS:8	AS:7	AS:6	**33**	**140**
*Erythrina lysistemon*	WH: 2	WH:3	WH:5	-	-	**10**	-	-	-	-	-	**0**	-	-	-	-	-	-	**0**	**10**
*Eucomis autumnalis*	FA:1”	-	-	-	-	**1**	-	-	-	-	-	**0**	-	-	-	-	-	-	**0**	**1**
	-	-	NC:8	-	-	**8**	-	-	-	-	-	**0**	-	-	-	-	-	-	**0**	**8**
*Eucomis pallidiflora*	-	-	-	FA: 9	-	**9**	-	-	-	-	-	**0**	-	-	-	-	-	-	**0**	**9**
	-	-	NC: 8	-	-	**8**	-	-	-	-	-	**0**	NC:2	-	NC:1	-	-	-	**3**	**11**
	-	-	-	-	-	**0**	-	-	-	WH:1	-	**1**	-	-	-	-	-	-	**0**	**1**
*Euphorbia schinzii*	-	-	-	-	-	**0**	AS:13	-	-	AS:6	-	**19**	-	-	-	-	-	-	**0**	**19**
*Gossypium herbaceum*	-	AS:8	AS:7	AS:11	AS:13	**39**	-	AS:4	AS:6	-	-	**10**	-	-	-	AS:8	AS:7	AS:6	**21**	**70**
*Grewia hispida*	-	-	NC:2	-	-	**2**	-	-	-	-	-	**0**	-	-	-	-	-	-	**0**	**2**
*Grewia sulcata*	-	-	-	-	-	**0**	-	-	-	-	AS:2	**2**	-	-	-	-	-	-	**0**	**2**
*Harpephyllum caffrum*	-	-	-	-	-	**0**	-	-	-	AS:1	-	**1**	-	-	-	-	-	-	**0**	**1**
*Helichrysum caespititium*	-	-	-	-	-	**0**	-	FA:1”	-	-	-	**1**	-	-	-	-	-	-	**0**	**1**
*Helichrysum gymnocomum*	-	-	AS: 2	-	-	**2**	-	-	-	-	-	**0**	-	-	-	-	-	-	**0**	**2**
*Hypoxis hemerocallidea*	-	-	-	AS:1	AS:12	**13**	-	AS:1”	-	-	-	**1**		-		-	-	-	**0**	**14**
	-	-	-	-	-	**0**	-	FA:4	-	-	-	**4**	-	-	-	-	-	-	**0**	**4**
*Hypoxis obtusa*	FA: 2	-	-	-	-	**2**	-	-	-	-	-	**0**	-	-	-	-	-	-	**0**	**2**
*Ipomoea albivenia*	-	-	-	-	-	**0**	-	-	-	-	AS:1	**1**	-	-	-	-	-	-	**0**	**1**
*Jatropha zeyheri*	AS:1	-	-	-	-	**1**	-	-	-	-	-	**0**	-	-	-	-	-	-	**0**	**1**
*Kalanchoe brachyloba*	-	-	NC:8	-	-	**8**	-	NC:1	NC:1	NC:2	NC:1	**5**	-	-	-	-	-	-	**0**	**13**
*Kirkia wilmsii*	-	-	-	-	-	**0**	-	-	-	-	-	**0**	-	-	-	AS:2	-	-	**2**	**2**
	-	-	NC:1	-	-	**1**	-	-	-	-	-	**0**	-	-	-	-	-	-	**0**	**1**
*Lantana rugosa*	-	-	-	-	-	**0**	-	-	-	-	-	**0**	-	-	-	AS:3	AS:1	AS:1	**5**	**5**
*Lasiosiphon caffer*	AS:14	AS:15	AS:13	AS:11	AS:15	**68**	AS:13	AS:10	AS:6	AS:6	AS:4	**39**	AS:7	AS:5	-	AS:8	AS:7	AS:6	**33**	**140**
	TC:5	TC:12	-	-	-	**17**	-	-	-	-	-	**0**	-	-	-	-	-	-	0	**17**
*Leonotis leonurus*	-	-	-	-	-	**0**	AS:3	AS:2	AS:2	AS:6	AS:1	**14**	-	-	-	-	-	-	**0**	**14**
*Lippia javanica*	-	-	-	-	-	**0**	-	-	-	-	-	**0**	-	-	-	-	AS:1”:3	AS:6	**10**	**10**
	-	-	-	-	-	**0**	-	FA:1”	-	-	-	**1**	-	-	-	-	-	-	**0**	**1**
	LB:2	-	-	-	-	**2**	-	-	-	-	-	**0**	-	-	-	-	-	-	**0**	**2**
	-	-	NC:1	-	-	**1**	-	-	-	-	-	**0**	-	-	-	-	-	-	**0**	**1**
	-	-	-	-	-	**0**	-	-	-	-	-	**0**	WH:1	-	-	WH:3	-	-	**4**	**4**
*Maerua juncea*	-	-	AS:3	-	-	**3**	-	-	-	-	-	**0**	-	-	-	-	-	-	**0**	**3**
	FA:1”	FA:2	-	-	-	**3**	-	-	-	-	-	**0**	-	-	-	-	-	-	**0**	**3**
*Mangifera indica*	-	-	-	-	-	**0**	-	-	-	-	-	**0**	-	AS:1	-	-	-	-	**1**	**1**
*Mimusops obovata*	AS:14	AS:15	AS:13	AS:11	AS:15	**68**	AS:13	AS:10	AS:6	AS:6	AS:4	**39**	AS:7	AS: 5	-	AS:8	AS:7	AS: 6	**33**	**140**
*Moringa oleifera*	FA:1	FA:7	FA:1	-	-	**9**	-	FA:3	FA:1	-	FA:4	**8**	-	-	-	-	-	-	**0**	**17**
*Olea europaea*	-	-	-	-	-	**0**	-	-	AS:1	-	AS:4	**5**	-	-		-	-	-	**0**	**5**
*Opuntia ficus-indica*	-	AS:1	-	-	-	**1**	-	-	-	-	-	**0**	-	-	-	-	-	-	**0**	**1**
*Osyris lanceolata*	-	-	AS:4	AS: 6	-	**10**	-	-	-	-	AS: 4	**4**	-	-		-			**0**	**14**
	-	-	-	-	-	**0**	-	FA:1”	-	-	-	**1**	-	-	-	-	-	-	**0**	**1**
	LB:1”	-	-	-	-	**1**	-	-	-	-	-	**0**	-	-	-	-	-	-	**0**	**1**
*Panica granatum*	-	-	-	-	-	**0**	-	-	AS:1	-	-	**1**	-	-	-	-	-	-	-	**1**
*Peltophorum africanum*	AS:1”	-	AS:1”	-	-	**2**	-	AS:1”	-	-	-	**1**	-	-	-	-	-	-	**0**	**3**
	-	FA:1”	-	FA:3	FA:1	**5**	-	-	-	FA:6	-	**6**	-	-	-	-	-	-	**0**	**11**
*Plumbago zeylanica*	-	-	-	-	-	**0**	-	-	-	-	-	**0**	AS:4	-	-	-	-	-	**4**	**4**
	-	-	-	-	-	**0**	NC:1	-	-	-	-	**1**	-	-	-	-	-	-	**0**	**1**
*Protea caffra*	-	-	AS:1	-	AS: 3	**4**	-	-	-	-	-	**0**	AS:1	-	-	-	AS:3	-	**4**	**8**
	-	-	-	-	-	**0**	FA:1	-	-	-	-	**1**	-	-	-	-	-	-	**0**	**1**
*Pseudognaphalium luteo-album*	-	-	-	-	-	**0**	FA:1	-	-	-	FA:1	**2**	-	-	-	-	-	-	**0**	**2**
*Psiadia punctulata*	-	-	AS:3	-	-	**3**	-	-	-	-	-	**0**	AS:1	-	-	AS: 3	-	-	**4**	**7**
	-	-	NC:6	-	-	**6**	-	-	-	-	-	**0**	-	-	-	-	-	-	**0**	**6**
*Ptaeroxylon obliquum*	-	-	-	-	AS:15	**15**	-	AS:10	-	-	-	**10**	-	-	-	-	-	-	**0**	**25**
*Pyrenacantha grandiflora*	-	-	-	-	-	**0**	AS:1”	-	-	-	-	**1**	-	-	-	-	-	-	**0**	**1**
*Rhoicissus tomentosa*	-	-	-	-	-	**0**	-	-	-		AS:4	**4**	-	-	-	-	-	-	**0**	**4**
*Schinus molle*	-	-	AS:1	-	-	**1**	-	-	-	-	-	**0**	-	-	-	-	-	-	**0**	**1**
	-	-	-	-	-	**0**	FA:3	-	-	-	FA:1	**4**	-	-	-	-	-	-	**0**	**4**
	-	-	LB:1			**1**	-	-	-	-	-	**0**	-	LB:1	-	-	-	-	**1**	**2**
*Schizoglossum nitidum*	-	-	-	-	-	**0**	-	AS:2	-	-	-	**2**	-	-	-	-	-	-	**0**	**2**
	-	-	NC:1	-	-	**1**	-	-	-	-	-	**0**	-	-	-	-	-	-	**0**	**1**
*Schkuhria pinnata*	-	-	-	-	-	**0**	AS:1”	-	-	-	-	**1**	-	-	-	-	-	-	**0**	**1**
*Sclerocarya birrea*	AS:1”:13	AS:15	AS:13	AS:11	AS:15	**68**	AS:13	AS:1”:9	AS:6	AS:6	AS:4	**39**	AS:7	AS:5	-	AS:8	AS:7	AS:6	**33**	**140**
*Securidaca longepedunculata*	-	-	-	-	-	**0**	-	-	-	-	-	**0**	AS:1	-	AS:1	-	-	-	**2**	**2**
*Senecio serratuloides*	-	-	AS:4	-	-	**4**	-	-	-	-	-	**0**	-	-	-	-	-	-	**0**	**4**
	-	-	-	-	-	**0**	-	FA:1	-	-	FA:3	**4**	-	-	-	-	-	-	**0**	**4**
	-	-	-	-	-	**0**	-	WH:4	-	-	-	**4**	-	-	-	-	-	-	**0**	**4**
*Senna italica*	-	AS:1	-	-	-	**1**	-	AS:1”	-	-	-	**1**	AS:7	-	-	-	-	-	**7**	**9**
*Sida cordifolia*	-	-	-	-	AS:3	**3**	-	-	-	-	-	**0**	-	-	-	-	-	-	**0**	**3**
*Siphonochilus aethiopicus*	-	-	-	-	-	**0**	-	AS:1”	-	-	-	**1**	-	-	-	-	-	-	**0**	**1**
*Solanum catombelense*	-	-	-	-	-	**0**	-	-	-	-	-	**0**	AS:1	AS:1	-	-	-	-	**2**	**2**
*Solanum panduriforme*	-	-	-	-	-	**0**			AS:1	-	AS:2	**3**	-	-	-	-	-	-	**0**	**3**
*Sorghum bicolor*	-	-	-	-	-	**0**	-	-	-	FA:6	-	**6**	-	-	-	-	-	-	**0**	**6**
*Strophanthus speciosus*	AS:10	-	-	-	AS:1	**11**	AS:2	-	-	-	-	**2**	AS:2	-	-	-	-	-	**2**	**15**
*Stylochaeton natalensis*	AS:14	AS:15	AS:13	AS:11	AS:15	**68**	AS:13	AS:10	AS:6	AS:6	AS:4	**39**	AS:7	AS:5	-	AS:8	AS:7	AS:6	**33**	**140**
*Tragia dioica*	-	-	-	-	-	**0**	-	FA:1”	-	-	-	**1**	-	-	-	-	-	-	**0**	**1**
*Tulbaghia violacea*	-	-	-	-	-	**0**	-	NC:2	-	-	-	**2**	-	-	-	-	-	-	**0**	**2**
*Vepris reflexa *	-	-	-	-	-	**0**	FA:1	FA:1	-	-	-	**2**	-	-	-	-	-	-	**0**	**2**
*Vernonia natalensis*	-	AS:3	-	-	AS:11	**14**	-	-	-	-	-	**0**	-	-	-	-	-	-	**0**	**14**
	-	-	NC:8	-	-	**8**	-	NC:1	NC:1	NC:2	NC:1	**5**	-	-	-	-	-	-	**0**	**13**
*Warburgia salutaris*	-	-	-	-	-	**0**	-	-	-	-	-	**0**	-	-	-	-	AS:4	-	**4**	**4**
	-	-	-	-	-	**0**	-	-	-	-	-	**0**	WH:7	-	-	-	-	-	**7**	**7**
*Withania somnifera*	-	-	-	-	-	**0**	-	-	-	FA:6	-	**6**	-	-	-	-	-	-	**0**	**6**
	LB:1”	-	-	-	-	**1**	-	-	-	-	-	**0**	-	-	-	-	-	-	**0**	**1**
*Xerophyta retinervis*	-	-	-	-	-	**0**	-	-	AS:3	AS:1	-	**4**	-	-	-	-	-	-	**0**	**4**
*Ximenia caffra*	AS:1”	-	AS:6	-	-	**7**	-	AS:1	-	-	-	**1**	-	-	-	-	-	-	**0**	**8**
*Zantedeschia aethiopica*	-	-	AS:7	-	-	**7**	-	-	-	-	-	**0**	-	-	-	-	-	-	**0**	**7**
*Zanthoxylum capense*	-	-	-	-	AS:1”	**1**	-	-	-	-		**0**	AS:1	-	-	-	AS:4	-	**5**	**6**
	-	-	WH:1	-	-	**1**	-	-	-	WH:6	-	**6**	-	-	-	-	-	-	**0**	**7**
*Zea mays*	-	-	-	-	-	**0**	-	-	-	-	-	**0**	-	-	-	-	AS:1	-	**1**	**1**
*Ziziphus mucronata*	AS:1	-	-	-	-	**1**	-	-	-	-	-	**0**	-	-	-	-	-	-	**0**	**1**

Fatigue: **FA**, laboured breathing: **LB**, nasal congestion: **NC**, and wheezing: **WH**. Plain numeric indicates number of healer/s who use a species to treat an ailments whilst numeric with a quotation mark indicates number of healer/s who use a species in combination to treat an ailment/s.

## Data Availability

The data used to support the findings of this study are available from the corresponding author upon request.

## References

[1] Castro H. J., Malka-Rais J., Bellanti J. A. (2005). Symposium: Current epidemiology of asthma: Emerging patterns of asthma. *Allergy and Asthma Proceedings*.

[2] Oni A. O., Erhabor G. E., Egbagbe E. E. (2010). The prevalence, management and burden of asthma - A nigerian study. *Iranian Journal of Allergy, Asthma and Immunology*.

[3] World Health Organisation (WHO) (2010). *Global initiative for asthma: Global strategy for asthma management and prevention*.

[4] Australian Bureau of Statistics, Profiles of Health, Australia: Asthma, 2011-2013, http://www.abs.gov.au, 2013

[5] Jindal S. K., Aggarwal A. N., Gupta D. (2012). Indian study on epidemiology of asthma, respiratory symptoms and chronic bronchitis in adults (INSEARCH). *The International Journal of Tuberculosis and Lung Disease*.

[6] Kahwa E. K., Waldron N. K., Younger N. O. (2012). Asthma and allergies in Jamaican children aged 2–17years: a cross-sectional prevalence survey. *BMJ Open*.

[7] Carlsen K. C. L., Haland G., Devulapalli C. S. (2006). Asthma in every fifth child in Oslo, Norway: A 10-year follow up of a birth cohort study. *Allergy: European Journal of Allergy and Clinical Immunology*.

[8] Nafti S., Taright S., El Ftouh M. (2009). Prevalence of asthma in North Africa: the Asthma Insights and Reality in the Maghreb (AIRMAG) study. *Respiratory Medicine*.

[9] Kirenga J. B., Okot-Nwang M. (2012). The proportion of asthma and patterns of asthma medications prescriptions among adult patients in the chest, accident and emergency units of a tertiary health care facility in Uganda. *African Health Sciences*.

[10] Marsden E. J., Somwe S. W., Chabala C., Soriano J. B., Vallès C. P., Ancochea J. (2016). Erratum to “Knowledge and perceptions of asthma in Zambia: A cross-sectional survey”, BMC Pulm Med. 2016;16:33. doi:10.1186/s12890-016-0195-3.. *BMC Pulmonary Medicine*.

[11] Masoli M., Fabian D., Holt S. S., Beasley R. (2003). *Global burden of asthma*.

[12] Bradshaw D., Nannan N., Laubscher R., Groenewald P., Joubert J., Nojilana B. (2004). South African national burden of disease study 2000: Estimates of provincial mortality. *South African Medical Research Council*.

[13] Bousquet J., Dahl R., Khaltaev N. (2007). Global alliance against chronic respiratory diseases. *Allergy*.

[14] Jackson S., Jansen P., Mangoni A. (2009). Prescribing for Elderly Patients. *Prescribing for Elderly Patients*.

[15] Ait-Khaled N., Enarson D. A., Bissell K., Billo N. E. (2007). Access to inhaled corticosteroids is key to improving quality of care for asthma in developing countries. *Allergy: European Journal of Allergy and Clinical Immunology*.

[16] Aït-Khaled N., Enarson D. A., Chiang C.-Y. (2008). COPD management. Part II. Relevance for resource-poor settings. *The International Journal of Tuberculosis and Lung Disease*.

[17] Noumi E. (2010). Ethno-medico-botanical survey of medicinal plants used in the treatment of asthma in the Nkongsamba Region, Cameroon. *Indian Journal of Traditional Knowledge*.

[18] Sonibare M. A., Gbile Z. O. (2008). Ethnobotanical survey of anti-asthmatic plants in South Western Nigeria. *African Journal of Traditional, Complementary and Alternative Medicines*.

[19] Asiimwe S. N., Borg-Karlsson A. K., Kamatenesi-Mugisha M., Oryem-Origa H. (2014). Documentation and consensus of indigenous knowledge on medicinal plants used by the local communities of western Uganda. *Journal of Natural Product and Plant Resources*.

[20] Amuka O., Okemo P. O., Machocho A. K., Mbugua P. K. (2014). Ethnobotanical survey of selected medicinal plants used by Ogiek communities in Kenya against microbial infections. *Ethnobotany Research and Applications*.

[21] Moteetee A., Van Wyk B.-E. (2011). The medical ethnobotany of Lesotho: a review. *Bothalia*.

[22] Motlhanka D., Nthoiwa G. P. (2013). Ethnobotanical Survey of medicinal plants of Tswapong North, in Eastern Botswana: a case of plants from Mosweu and Seolwane Villages. *European Journal of Medicinal Plants*.

[23] Hutchings A., H Scott A., Lewis G., Cunningham A. B. (1996). *Zulu Medicinal Plants: An inventory*.

[24] Thring T. S. A., Weitz F. M. (2006). Medicinal plant use in the Bredasdorp/Elim region of the Southern Overberg in the Western Cape Province of South Africa. *Journal of Ethnopharmacology*.

[25] De Beer J. J. J., Van Wyk B.-E. (2011). An ethnobotanical survey of the Agter-Hantam, Northern Cape Province, South Africa. *South African Journal of Botany*.

[26] York T., De Wet H., Van Vuuren S. F. (2011). Plants used for treating respiratory infections in rural Maputaland, KwaZulu-Natal, South Africa. *Journal of Ethnopharmacology*.

[27] Bhat R. B. Plants of Xhosa people in the Transkei region of Eastern Cape (South Africa) with major pharmacological and therapeutic properties. *Journal of Medicinal Plants Research*.

[28] Perret S., Anseeuw W., Mathebula N. (2005). *Poverty and livelihoods in rural South Africa. Investigating diversity and dynamics of livelihoods. Case studies in Limpopo*.

[29] Mogawane M. A., Mothiba T. M., Malema R. N. (2015). Indigenous practices of pregnant women at Dilokong hospital in Limpopo province, South Africa. *Curationis*.

[30] Wow (2016). *Limpopo demography*.

[31] Al-Quran S. (2009). Ethnopharmacological survey of wild medicinal plants in Showbak, Jordan. *Journal of Ethnopharmacology*.

[32] Friedman J., Yaniv Z., Dafni A., Palewitch D. (1986). A preliminary classification of the healing potential of medicinal plants, based on a rational analysis of an ethnopharmacological field survey among Bedouins in the Negev Desert, Israel. *Journal of Ethnopharmacology*.

[33] Phillips O., Gentry A. H. (1993). The useful plants of Tambopata, Peru: II. additional hypothesis testing in quantitative ethnobotany. *Economic Botany*.

[34] Kuldip S. D., Sandeep C., Jeewan S. J. (2015). Assessment of Indian medicinal plants for the treatment of asthma. *Journal of Medicinal Plants Research*.

[35] Shankar R., Lavekar G. S., Deb S., Sharma B. K. (2012). Traditional healing practice and folk medicines used by Mishing community of North East India. *Journal of Ayurveda and Integrative Medicine*.

[36] Ojewole J. A. O. (2003). Evaluation of the anti-inflammatory properties of Sclerocarya birrea (A. Rich.) Hochst. (family: Anacardiaceae) stem-bark extracts in rats. *Journal of Ethnopharmacology*.

[37] Hossan M. S., Hanif A., Agarwala B. (2010). Traditional use of medicinal plants in Bangladesh to treat urinary tract infections and sexually transmitted diseases. *Ethnobotany Research and Applications *.

[38] Vaijanathappa J., Badami S., Bhojraj S. (2008). In vitro antioxidant activity of Enicostemma axillare. *Journal of Health Science*.

[39] Dzeufiet Djomeni P. D., Ngeutse Donfouet F., Dimo T. (2009). In vivo and in vitro antiasthmatic studies of Clerodendrum serratum Linn. *Pharmacologyonline*.

[40] Nonjinge S., Tarr B. B. (2013). *Natal National Botanical Garden*.

[41] Gelfand M., Mavi S., B Drummond R., Ndemera B. (1985). *The Traditional Medical Practitioner in Zimbabwe*.

[42] Maroyi A. (2008). Ethnobotanical study of two threatened medicinal plants in Zimbabwe. *International Journal of Biodiversity Science and Management*.

[43] South Africa National Biodiversity Institute (SANBI) (2016). *The IUCN Categories and Criteria version 3.1. SANBI*.

[44] Van der Merwe M. M. (2008). *Bioactive sesquiterpenoids from Dicoma anomala subsp. gerrardii*.

[45] Nzue A. P. M. (2009). *Use and conservation status of medicinal plants in the Cape Peninsula, Western Cape Province of South Africa*.

[46] Long C. (2005). *Swaziland's Flora-Siswati names and uses*.

[47] Coopoosamy R. M., Naidoo K. K. (2012). An ethnobotanical study of medicinal plants used by traditional healers in Durban, South Africa. *African Journal of Pharmacy and Pharmacology*.

[48] Van Wyk B.-E. (2008). A broad review of commercially important southern African medicinal plants. *Journal of Ethnopharmacology*.

[49] Nkosi S. E. (2014). *A vegetation classification and management plan for the Nooitgedacht section of the Loskop Dam Nature Reserve*.

[50] Motlhatlego E. M. (2014). *Evaluation of plants used in African traditional medicine for asthma and related conditions*.

[51] Suliman S. (2011). *Antimicrobial interaction of Artemisia afra used in African traditional medicine*.

[52] Watt J. M., Breyer-Brandwijk M. G. *The Medicinal and Poisonous Plants of Southern and Eastern Africa: Pharmacological Effects and Toxicology in Man and Animals*.

[53] Abubakar M. M. (2010). Antibacterial potential of crude leaf extracts of Eucalyptus camaldulensis against some pathogenic bacteria. *African Journal of Plant Science*.

[54] Nwinuka N. M., Monanu M. O., Nwiloh B. I. (2008). Effects of aqueous extract of Mangifera indica L. (Mango) stem bark on haematological parameters of normal albino rats. *Pakistan Journal of Nutrition*.

[55] Akah P. A., Gamaniel K. S., Samson A., Wambebe C. O. (1997). Evaluation of Nigerian traditional medicine: Effects of Gakani, a herbal anti-asthmatic drug. *Journal of Ethnopharmacology*.

[56] Loffler L., Loffler P. (2005). Swaziland Tree Atlas-including selected shrubs and climbers. *Southern African Botanical Diversity Network Report*.

[57] Emeagwali G., Dei G. J. S. (2014). *African Indigenous Knowledge and the Disciplines*.

[58] Khaleegur R., Arshiya S., Shafeequr R. (2012). Gossypium herbaceum Linn: An ethnopharmacological review. *Journal of pharmaceutical and Scientific Innovation*.

[59] Chinou I. (2012). *Assessment report on Olea europaea L., folium*.

[60] Osuna-Martínez U., Reyes-Esparza J., Rodríguez-Fragoso L. (2014). Cactus (*Opuntia ficus-indica*): a review on its antioxidants properties and potential pharmacological use in chronic diseases. *Natural Products Chemistry & Research*.

[61] Sunil A., Dhasade V., Patil M., Pal S., Subhash C., Barwal S. (2009). Antihistaminic effect of various extracts of. *Journal of Young Pharmacists*.

[62] Sharma D., Soni M., Kumar S., Gupta G. D. (2009). Solubility enhancement##hssm###8212;eminent role in poorly soluble drugs. *Research Journal of Pharmacy and Technology*.

[63] Shikhakhane X. (2013). *Evaluating anticancer and antimicrobial properties of extracts from hypoxis hemerocallidea (African potatoe). M.Sc. dissertation [dissertation, thesis]*.

[64] Mills E., Cooper C., Seely D., Kanfer I. (2005). African herbal medicines in the treatment of HIV: Hypoxis and Sutherlandia. An overview of evidence and pharmacology. *Nutrition Journal *.

[65] Schumacher T. N., Schreiber R. D. (2015). Neoantigens in cancer immunotherapy. *Science*.

[66] Ncube B., Ndhlala A. R., Okem A., Van Staden J. (2013). Hypoxis (Hypoxidaceae) in African traditional medicine. *Journal of Ethnopharmacology*.

[67] Ojewole J., Kamadyaapa D. R., Musabayane C. T. (2006). Some in vitro and in vivo cardiovascular effects of Hypoxis hemerocallidea Fisch & CA Mey (Hypoxidaceae) corm (African potato) aqueous extract in experimental animal models. *Cardiovascular Journal of Africa*.

[68] Chaudhari S. S., Chaudhari G. S. (2015). A review on plumbago zeylanica linn. - A divine medicinal plant. *International Journal of Pharmaceutical Sciences Review and Research*.

[69] Keriko J. M., Nakajima S., Baba N., Iwasa J. (1997). Eicosanyl p-coumarates from a kenyan plant, psiadia punctulata: Plant growth inhibitors. *Bioscience, Biotechnology, and Biochemistry*.

[70] Van Wyk B. E., Gericke N. (2000). *Peoples Plants. Briza Publications*.

[71] Kayani S., Ahmad M., Zafar M. (2014). Ethnobotanical uses of medicinal plants for respiratory disorders among the inhabitants of Gallies–Abbottabad, Northern Pakistan. *Journal of Ethnopharmacology*.

[72] Encyclopedia Memim. http://memim.com/warburgia-salutaris.html.

[73] (2006). *Developing treatments*.

[74] Helito C. P., Demange M. K., Bonadio M. B. (2013). Anatomy and histology of the knee anterolateral ligament. *Orthopaedic Journal of Sports Medicine*.

[75] Das B. C., Marappan G., Saha S., Bhowmik D., Chiranjib (2010). Anthelmintic and anti-microbial activity of some novel chalcone derivatives. *Journal of Chemical and Pharmaceutical Research*.

[76] Mukinda J. T. (2007). *Acute and chronic toxicity of the flavonoid-containing plant, Artemisia afra in rodents [dissertation, thesis]*.

[77] Morobe I. C., Mthethwa N. S., Bisi-Johnson M. A. (2012). Cytotoxic effects and safety profiles of extracts of active medicinal plants from South Africa. *Journal of Microbiology Research*.

[78] Ndhlala A. R., Ncube B., Okem A., Mulaudzi R. B., Van Staden J. (2013). Toxicology of some important medicinal plants in southern Africa. *Food and Chemical Toxicology*.

[79] Fawole O. A., Amoo S. O., Ndhlala A. R., Light M. E., Finnie J. F., Van Staden J. (2010). Anti-inflammatory, anticholinesterase, antioxidant and phytochemical properties of medicinal plants used for pain-related ailments in South Africa. *Journal of Ethnopharmacology*.

[80] Hammond G. B., Fernández I. D., Villegas L. F., Vaisberg A. J. (1998). A survey of traditional medicinal plants from the Callejon de Huaylas, Department of Ancash, Peru. *Journal of Ethnopharmacology*.

[81] B-. Van Wyk E., Van Oudtshoorn B., Gericke N. (1997). *Medicinal Plants of South Africa*.

[82] Dlamini T. P. (2006). *Isolation and characterisation of bio-active compounds from Lippia javanica*.

[83] Wickens G. E., Kunkel G. (1979). The uses of the baobab (Adansonia digitata L.) in Africa. *Taxonomic aspects of African economic botany*.

[84] Kalix P. (1996). Catha edulis, a plant that has amphetamine effects. *Pharmacy world and science*.

[85] Mehta J., Shukla A., Bukhariya V., Charde R. (2011). THE MAGIC REMEDY OF MORINGA OLIFERIA: AN OVERVIEW. *International Journal of Biomedical and Advance Research*.

[86] Njoroge G. N., Bussmann R. W. (2006). Traditional management of ear, nose and throat (ENT) diseases in Central Kenya. *Journal of Ethnobiology and Ethnomedicine*.

[87] Umadevi M., Rajeswari R., Rahale C. S. (2012). Traditional and medicinal uses of Withania Somnifera. *The Pharma Innovation*.

[88] Semenya S. (2012). *Bapedi phytomedicine and their use in the treatment of sexually transmitted diseases in Limpopo Province, South Africa*.

[89] NIR Board of Consultants and Engineers, Cultivation and Processing of Selected Medicinal Plants, Asoa Pacific Business Press Inc, Delhi, India, 2006

[90] Semenya S. S., Potgieter M. J., Erasmus L. J. C. (2013). Indigenous plant species used by Bapedi healers to treat sexually transmitted infections: Their distribution, harvesting, conservation and threats. *South African Journal of Botany*.

